# Biallelic Mutations in *KDSR* Disrupt Ceramide Synthesis and Result in a Spectrum of Keratinization Disorders Associated with Thrombocytopenia

**DOI:** 10.1016/j.jid.2017.06.028

**Published:** 2017-11

**Authors:** Takuya Takeichi, Antonio Torrelo, John Y.W. Lee, Yusuke Ohno, María Luisa Lozano, Akio Kihara, Lu Liu, Yuka Yasuda, Junko Ishikawa, Takatoshi Murase, Ana Belén Rodrigo, Pablo Fernández-Crehuet, Yoichiro Toi, Jemima Mellerio, José Rivera, Vicente Vicente, David P. Kelsell, Yutaka Nishimura, Yusuke Okuno, Daiei Kojima, Yasushi Ogawa, Kazumitsu Sugiura, Michael A. Simpson, W.H. Irwin McLean, Masashi Akiyama, John A. McGrath

**Affiliations:** 1 St. John’s Institute of Dermatology, King’s College London (Guy’s Campus), London, UK; 2 Department of Dermatology, Nagoya University Graduate School of Medicine, Nagoya, Japan; 3 Department of Dermatology, Hospital Infantil del Niño Jesús, Madrid, Spain; 4 Faculty of Pharmaceutical Sciences, Hokkaido University, Sapporo, Japan; 5 Centro Regional de Hemodonación, Servicio de Hematología y Oncología Médica, Hospital Universitario Morales Meseguer, IMIB-Arrixaca, Universidad de Murcia, Centro de Investigación Biomédica en Red de Enfermedades Raras, Instituto de Salud Carlos III, Madrid, Spain; 6 Viapath, St. Thomas’ Hospital, London, UK; 7 Analytical Science Research Laboratories, Kao Corporation, Haga, Tochigi, Japan; 8 Biological Science Research Laboratories, Kao Corporation, Haga, Tochigi, Japan; 9 Department of Dermatology, Hospital Sierra de Segura, Puente de Génave, Jaén, Spain; 10 Department of Dermatology, Hospital Universitario Reina Sofía, Córdoba, Spain; 11 Department of Dermatology, Hiroshima City Hiroshima Citizens Hospital, Hiroshima, Japan; 12 Department of Dermatology, Great Ormond Street Hospital for Children NHS Foundation Trust, London, UK; 13 Centre for Cell Biology and Cutaneous Research, Blizard Institute, Barts, London, UK; 14 London School of Medicine and Dentistry, Queen Mary University of London, Whitechapel, London, UK; 15 Department of General Perinatology, Hiroshima City Hiroshima Citizens Hospital, Hiroshima, Japan; 16 Center for Advanced Medicine and Clinical Research, Nagoya University Hospital, Nagoya, Japan; 17 Department of Pediatrics, Nagoya University Graduate School of Medicine, Nagoya, Japan; 18 Department of Dermatology, Fujita Health University School of Medicine, Toyoake, Japan; 19 Department of Medical and Molecular Genetics, King’s College London, School of Medicine, Guy’s Hospital, London, UK; 20 Centre for Dermatology and Genetic Medicine, Division of Molecular Medicine, University of Dundee, Dundee, UK

**Keywords:** DHS, dihydrosphingosine, KDS, 3-ketodihydrosphingosine, S1P, sphingosine-1-phosphate

## Abstract

Mutations in ceramide biosynthesis pathways have been implicated in a few Mendelian disorders of keratinization, although ceramides are known to have key roles in several biological processes in skin and other tissues. Using whole-exome sequencing in four probands with undiagnosed skin hyperkeratosis/ichthyosis, we identified compound heterozygosity for mutations in *KDSR*, encoding an enzyme in the de novo synthesis pathway of ceramides. Two individuals had hyperkeratosis confined to palms, soles, and anogenital skin, whereas the other two had more severe, generalized harlequin ichthyosis-like skin. Thrombocytopenia was present in all patients. The mutations in *KDSR* were associated with reduced ceramide levels in skin and impaired platelet function. KDSR enzymatic activity was variably reduced in all patients, resulting in defective acylceramide synthesis. Mutations in *KDSR* have recently been reported in inherited recessive forms of progressive symmetric erythrokeratoderma, but our study shows that biallelic mutations in *KDSR* are implicated in an extended spectrum of disorders of keratinization in which thrombocytopenia is also part of the phenotype. Mutations in *KDSR* cause defective ceramide biosynthesis, underscoring the importance of ceramide and sphingosine synthesis pathways in skin and platelet biology.

## Introduction

The hereditary palmoplantar keratodermas and ichthyoses comprise a heterogeneous collection of genodermatoses caused by mutations in more than 100 genes involved in a multitude of biologic pathways and processes (Oji et al., 2010, Sakiyama and Kubo, 2016). Despite major advances in discovering the underlying molecular genetic basis of many of these disorders, several cases remain unresolved, indicating the likely contribution of further gene pathology (Fischer, 2009).

One very recent discovery that expands the molecular pathology of ichthyosis has been the identification of mutations in *KDSR* in four individuals with clinical phenotypes of progressive symmetric erythrokeratoderma (Boyden et al., 2017). *KDSR* encodes 3-ketodihydrosphingosine reductase, which catalyzes the reduction of 3-ketodihydrosphingosine (KDS) to dihydrosphingosine (DHS), a key step in the de novo ceramide synthesis pathway (Linn et al., 2001). Previously, mutations in a different gene in this pathway, *CERS3,* have also been implicated in autosomal recessive congenital ichthyosis, emphasizing the clinical relevance of ceramide pathology in inherited disorders of cornification (Eckl et al., 2013, Radner et al., 2013). Ceramides also have key physiological roles in other organs: mutations in *ELOVL4*, encoding an enzyme necessary for the production of ultra-long chain ceramides in the skin, brain, and retina, lead to a recessive disorder characterized by ichthyosis, intellectual disability, and spastic quadriplegia (Aldahmesh et al., 2011).

In this study, we investigated four individuals from Spain, Japan, and the United Kingdom who presented with inherited disorders of keratinization but had clinical features different from those presented by Boyden et al. (2017). Two patients displayed a milder phenotype of palmoplantar and anogenital hyperkeratosis, whereas the other two patients had a more severe phenotype resembling harlequin ichthyosis. An additional finding, present in all our subjects, but not featured in the Boyden et al. study, was a reduction in the number of blood platelets (thrombocytopenia).

Using whole-exome sequencing, functional studies on skin and platelets, and in vitro analyses, we identified autosomal recessive mutations in *KDSR* in all four subjects, with only one heterozygous mutation overlapping with published findings (Boyden et al., 2017). Our findings expand the molecular and clinical pathology associated with *KDSR* mutations and show that this ceramide biosynthesis pathway has important roles in both skin and platelets.

## Results

### Clinical features of individuals with *KDSR* mutations

Permission to report medical details and include clinical illustrations was obtained for all patients (from guardians for patients 1, 3, and 4 and from patient 2 himself).

**Figure 1:**
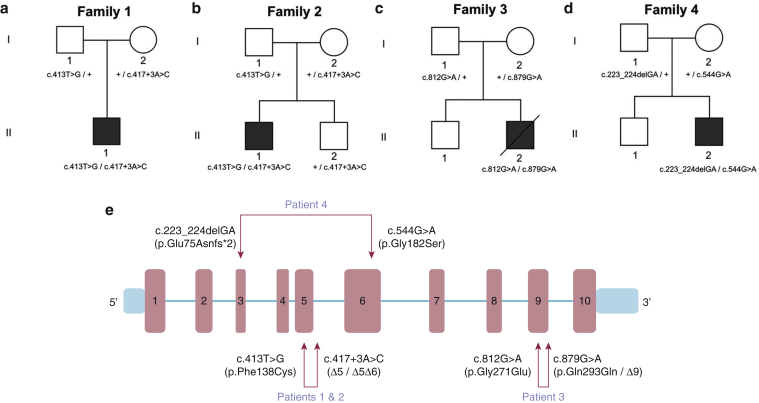
**Pedigrees and mutations identified in *KDSR*.** (**a–d**) Family pedigrees of the four patients with compound heterozygous mutations in *KDSR*. + denotes the wild-type allele. (**e**) Schematic of *KDSR* to show the six compound heterozygous mutations identified in this study.

**Figure 2:**
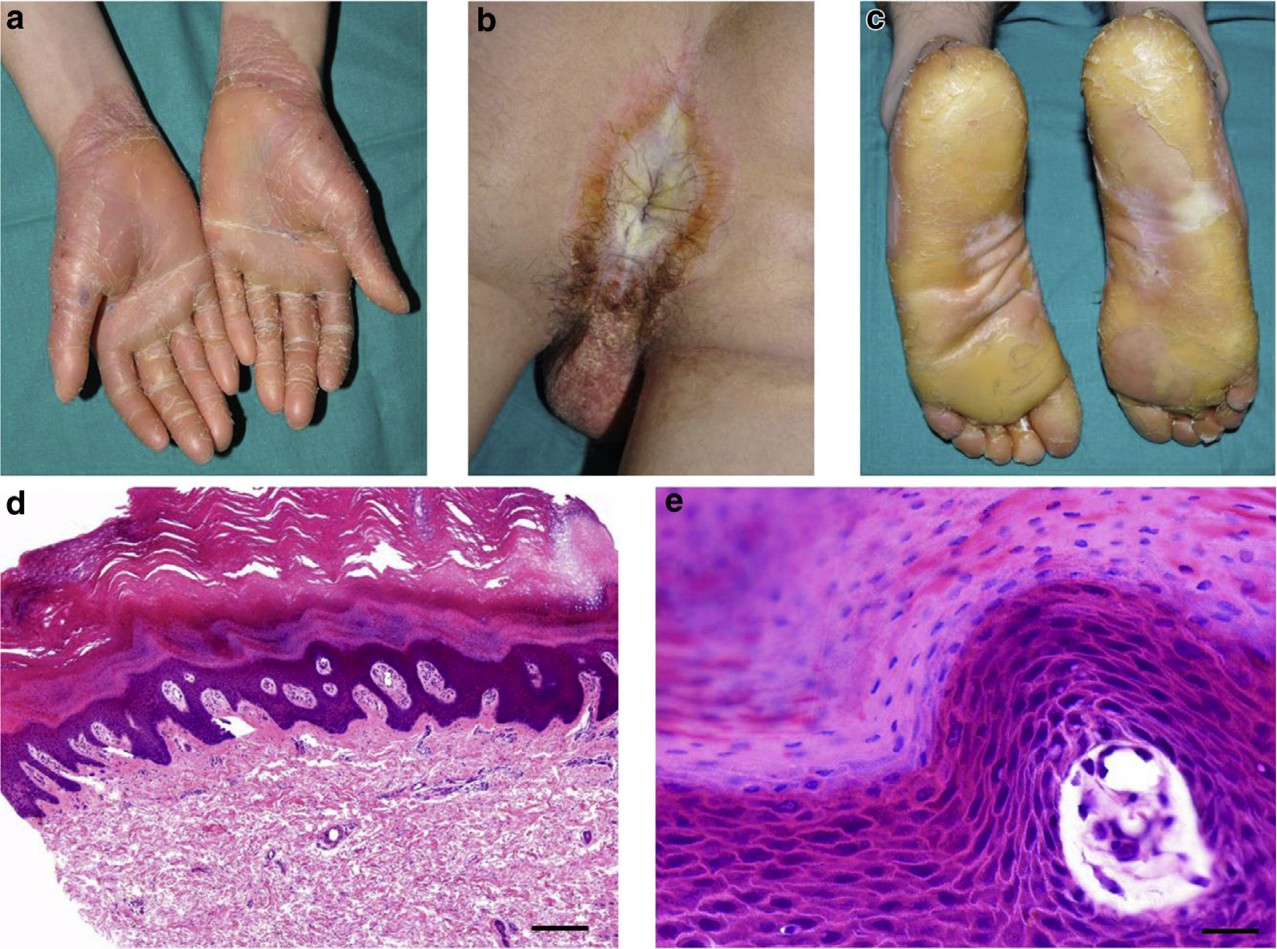
**Clinicopathologic features of patient 1.** (**a**) Diffuse palmar keratoderma. (**b**) Perianal hyperkeratosis. (**c**) Bilateral diffuse plantar keratoderma. (**d**) Light microscopy of palmar skin shows psoriasiform acanthosis and hyperkeratosis. Hematoxylin and eosin stain. Scale bar = 100 μm. (**e**) Higher magnification shows focal hypergranulosis and parakeratosis. Hematoxylin and eosin stain. Scale bar = 10 μm.

Patient 1 is a 15-year-old male and the only child of unrelated healthy parents (family 1, Figure 1a). His parents are originally from the same geographic area in mid-southeast Spain. At the age of 12 months, he developed palmoplantar hyperkeratosis with extension to the dorsae of the hands and feet, wrists, and ankles, as well as anogenital hyperkeratosis and erythema (Figure 2a–c). At age 2 years, a blood count was performed because of mucocutaneous bleeding, which showed a severe, isolated thrombocytopenia (platelet count < 30 × 10^9^/L; bone marrow biopsy sample showed a normal to increased number of megakaryocytes only). A diagnosis of primary immune thrombocytopenia was made, but treatment with oral corticosteroids was suboptimal. Splenectomy at age 11 years led to a slight increase in platelets (∼40 × 10^9^/L), although clinically he continues to suffer recurrent nose bleeds. Light microscopy of lesional skin showed nonspecific findings of psoriasiform acanthosis, parakeratosis, and focal hypergranulosis but no epidermolytic changes (Figure 2d and e). Oral acitretin (0.5 mg/kg) prescribed for several months did not lead to any improvement in his skin.

Patient 2 is a 21-year-old male and the older of two brothers born to healthy unrelated parents (family 2, Figure 1b). He is the only affected individual among his relatives. This family originates from the same geographic region in Spain as family 1. At age 15 months, he developed diffuse hyperkeratosis on the palms and soles, without progression to the dorsae of the hands or feet (i.e., less severe than patient 1). He also developed perianal erythema and hyperkeratosis. As for patient 1, oral acitretin did not improve the hyperkeratosis. In addition, he suffered episodes of bruising with evidence of isolated thrombocytopenia. Bone marrow studies showed normal hematologic morphology. At present, he has not manifested clinically relevant signs of bleeding despite persistently low platelets (∼20 × 10^9^/L).

Patient 3 was the second child born to unrelated white parents from the United Kingdom (family 3, Figure 1c). His parents, older brother, and all other relatives were healthy. His mother’s pregnancy was uneventful until the last trimester, when oligohydramnios was noted. She had spontaneous rupture of membranes at 33+5 weeks and underwent elective cesarian section at 35+2 weeks with an infant birth weight of 2.74 kg. At birth, the patient was covered in thick adherent plate-like scales, with prominent ectropion and eclabium, and pinching of all digits, collectively consistent with harlequin ichthyosis. He was treated in a humidified incubator with hourly greasy emollients and lubricating eye drops. Acitretin was started, which led to some reduction in adherent scaling, although he developed pseudomonas septicemia at age 15 days and further sepsis thereafter. At birth, platelet count was 120 × 10^9^/L, but within 2 weeks this dropped to 50 × 10^9^/L, and by the 3rd week to approximately 20–30 × 10^9^/L and remained at this level. At day 36, he deteriorated clinically with tachypnea and hypotension associated with a profound metabolic acidosis. Despite efforts to resuscitate him, he died age 37 days.

Patient 4 is a 6-year-old Japanese male and is the younger of two brothers born to unrelated parents (family 4, Figure 1d). His mother and brother have atopic dermatitis, but there is no other noteworthy family history. He was delivered at 35+3 weeks by normal spontaneous vaginal birth with a birth weight of 1.9 kg. At birth, he had thick plate-like scales with deep fissuring overlying erythrodermic skin. Severe eclabium and ectropion were also observed. Skin biopsy showed marked hyperkeratosis with parakeratosis (see Supplementary Figure S1 online). These features were consistent with Harlequin ichthyosis. He was treated in the neonatal intensive care unit but did not receive systemic retinoids. Over the first 2 months of life, the thick scales desquamated gradually, resulting in generalized erythroderma and fine scaling. Platelet count was normal at birth (140–150 × 10^9^/L), but since the age of 2 months this progressively decreased, and at 3 years of age he had severe thrombocytopenia (4–11 × 10^9^/L).

### Identification of compound heterozygous mutations in *KDSR* in all affected individuals

After ethics committee approval and written informed consent, whole-exome sequencing was performed using DNA from all affected probands. Candidate gene mutations were prioritized by filtering for variants with a frequency of less than 0.1% in public databases such as the Exome Aggregation Consortium (ExAC), Exome Variant Server, 1000 Genomes Project, and an in-house repository. Whole-exome sequencing showed compound heterozygous mutations in *KDSR*, a candidate gene recently implicated in progressive symmetric erythrokeratoderma (Boyden et al., 2017). Details of the mutations are shown in Figure 1e and Table 1; only one of the heterozygous mutant alleles overlapped with the known *KDSR* mutations. The mutations were verified by Sanger sequencing (see Supplementary Figure S2 online) and segregated with disease status in family members whose DNA was available (Figures 1a–d). Our study showed three missense mutations (p.Phe138Cys, p.Gly182Ser, and p.Gly271Glu), one synonymous variant (c.879G>A, p.Gln293Gln, but affecting the last base of an exon and therefore potentially a donor splice site mutation), one other splice site mutation (c.417+3G>A), and one out-of-frame deletion (c.223_224delGA, p.Glu75Asnfs*2) (Figure 1e). Patients 1 and 2 come from the same region of Spain, and therefore, although neither family was aware of any relatedness, the finding of identical compound heterozygotes mutations in *KDSR* (p.Phe138Cys and c.417+3G>A) is likely to indicate sharing of regional founder mutations. The splicing mutation (c.417+3A>C) was predicted to cause a reduction of 41.8% of transcripts expressing exon 5 of *KDSR*, based on the SPANR tool (Xiong et al., 2015), which was confirmed by reverse transcription-PCR using RNA extracted from skin (patients 1 and 2). Sequencing of cDNA from exon 2 to exon 7 of *KDSR* showed skipping of exon 5 (96 base pairs, Δ5) or skipping of exons 5 and 6 (288 base pairs, Δ5Δ6) (see Supplementary Figure S3a and b online). Both of these truncated transcripts restore the reading frames. By using cDNA from peripheral blood, in-frame skipping of exon 5 was shown (see Supplementary Figure S3c). The synonymous c.879G>A mutation (p.Gln293Gln) was previously identified in the study by Boyden et al. (2017). This nucleotide transition occurs within the last base of exon 9, and reverse transcription-PCR in that report showed in-frame skipping of exon 9.

**Table 1:** Summary of clinical and mutation details of all four affected individuals

Patient	Country of Origin	Dermatologic Phenotype	Thrombocytopenia	Mutations in *KDSR* and Amino Acid Change	1000 Genomes Project Frequency	ExAC Frequency	SIFT (Score)	PolyPhen-2 (Score)	Mutation Taster
1	Spain	Palmoplantar and perianal keratoderma	+	c.413T>G: p.Phe138Cys	0	8.3 × 10^–5^	Damaging	Probably damaging	Disease-causing
				c.417+3A>C	0	0	N/A	N/A	Disease-causing
2	Spain	Palmoplantar and perianal keratoderma	+	c.413T>G: p.Phe138Cys	0	8.3 × 10^–5^	Damaging	Probably damaging	Disease-causing
				c.417+3A>C	0	0	N/A	N/A	Disease-causing
3	United Kingdom	Harlequin ichthyosis	+	c.812G>A: p.Gly271Glu	0	0	Damaging	Probably damaging	Disease-causing
				c.879G>A: p.Gln293Gln	0	3.3 × 10^–5^	N/A	N/A	Disease-causing
4	Japan	Harlequin ichthyosis	+	c.223_224delGA: p.Glu75Asnfs*2	0	0	N/A	N/A	Disease-causing
				c.544G>A: p.Gly182Ser	0	8.2 × 10^–6^	Damaging	Probably damaging	Disease-causing

Abbreviations: ExAC, Exome Aggregation Consortium; N/A, not applicable; PolyPhen-2, Polymorphism Phenotyping v2; SIFT, Sorting Tolerant From Intolerant.

### *KDSR* mutations impair enzymatic activity and lead to defective acylceramide synthesis

**Figure 3:**
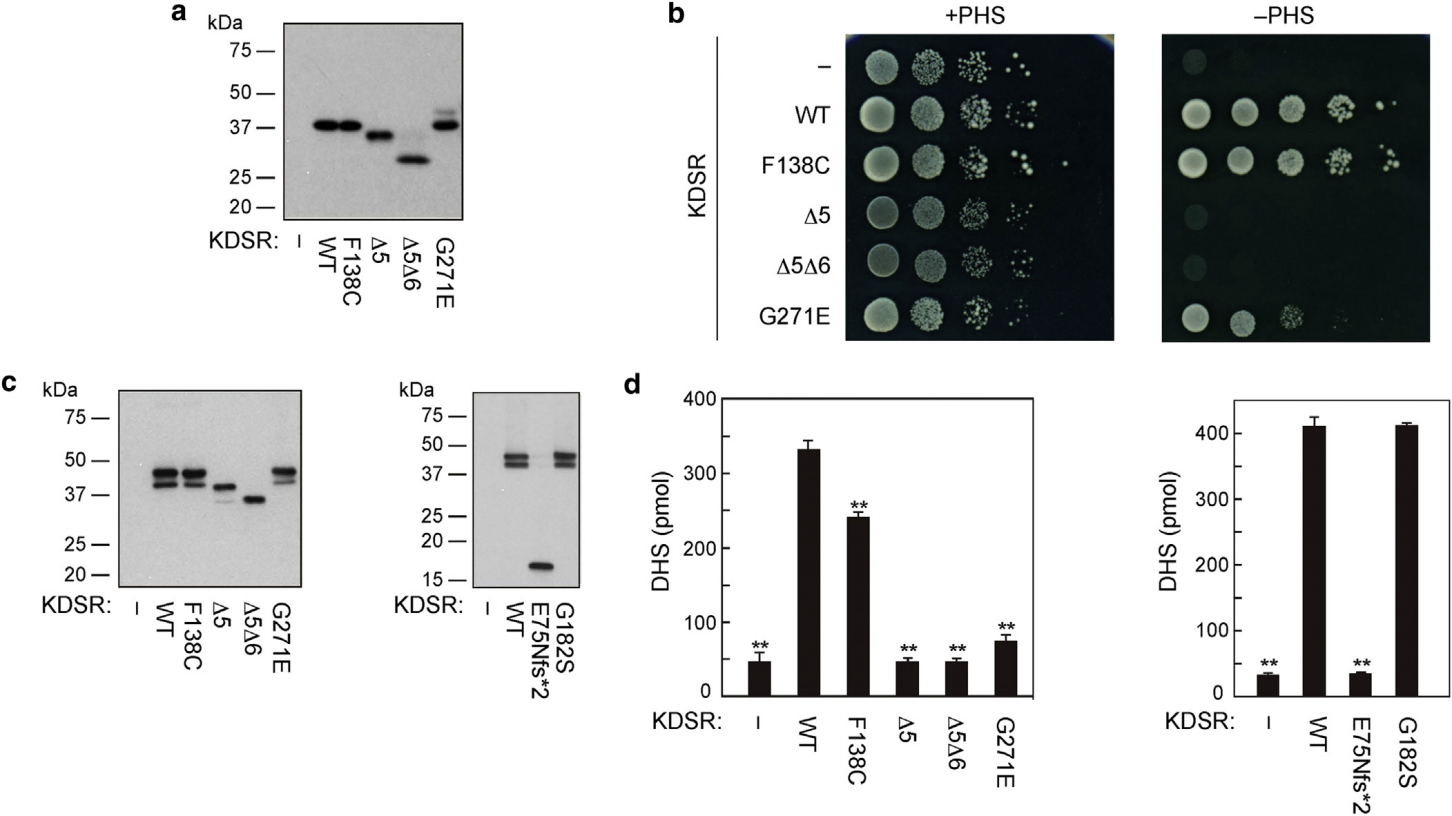
**Mutations in *KDSR* impair enzymatic activity in vitro.** (**a**) Total lysates prepared from KHY625 (*Δtsc10*) cells harboring an empty vector or the plasmid encoding WT or mutant *3xFLAG-KDSR* were separated by SDS-PAGE and subjected to immunoblotting using anti-FLAG M2 antibody. (**b**) KHY625 cells bearing the indicated plasmid were grown serially diluted at 1:10, spotted on SC-URA plates with or without 5 μmol/L PHS, and grown at 30 °C for 3 days. (**c, d**) HEK 293T cells were transfected with an empty vector or the plasmid encoding WT or mutant *3xFLAG-KDSR*. DHS levels in the membrane fractions from the cells transfected with empty vector are due to activity of endogenous KDSR. At 24 hours after transfection, total membrane fractions were prepared. (**c**) Total membrane fractions (5 μg protein) were separated by SDS-PAGE and subjected to immunoblotting using anti-FLAG M2 antibody. (**d**) Total membrane fractions were incubated with 10 μmol/L KDS and 1 mmol/L NADPH at 37 °C for 1 hour. Lipids were extracted and subjected to liquid chromatography-mass spectrometry/mass spectrometry analysis. DHS was detected in the MRM mode and quantified using MassLynx software. Values represent the mean ± standard deviations of three independent experiments. Statistically significant differences compared with WT are indicated. ^∗∗^*P* < 0.01; Tukey test. DHS, dihydrosphingosine; KDS, 3-ketodihydrosphingosine; KDSR, 3-ketodihydrosphingosine reductase; MRM, multiple reaction monitoring; NADPH, nicotinamide adenine dinucleotide phosphate; PHS, phytosphingosine; SC-URA, synthetic complete minus uracil; WT, wild type.

To assess the effect of the mutations on KDSR enzymatic activity, the new mutations identified in our patients were introduced into yeast and HEK 293T cells. Two mutant plasmids were designed for the c.417+3A>C variant, one predicting skipping of exon 5 only (Δ5) and the other loss of both exons 5 and 6 (Δ5Δ6) (Figure 3a). Primer details for the mutant constructs are shown in Supplementary Table S1 online.

A yeast complementation assay was performed using yeast grown on plates with or without phytosphingosine. Because sphingolipids are essential for cell viability, Δ*tsc10* yeast cells cannot grow normally unless the addition of phytosphingosine or DHS to the medium bypasses the requirement of de novo sphingolipid synthesis. Therefore, under these circumstances, yeast would not be able to grow normally if the *KDSR* mutants impair enzymatic activity. This assay showed that the mutations (illustrated for patients 1, 2, and 3; Figure 3b) diminished the ability of yeast to grow in the absence of phytosphingosine, similar to the three different mutations tested by Boyden et al. (2017). The p.Phe138Cys mutant had the mildest effect. In comparison, the Δ5 and Δ5Δ6 mutants (representing the c.417+3A>C mutation) resulted in the most significant impairment of yeast growth (Figure 3b).

To assess the enzymatic activity in vitro, all mutant constructs (including those for patient 4, performed separately under similar conditions) were introduced into HEK 293T cells (Figure 3c), and KDSR activity was measured in vitro using membrane fractions, notwithstanding an inherent limitation of this assay being that HEK 293T cells have endogenous KDSR activity, which is not abolished. Consistent with the yeast complementation assay, this showed that most of the mutants led to a significant reduction in DHS synthesis (Figure 3d). The only exception was the p.Gly182Ser (c.544G>A) variant, which showed no significant difference in DHS synthesis compared with the wild type (Figure 3d). The p.Gly182Ser mutation occurs within the hydrophilic domain (amino acids 22–270), similar to two mutations found by Boyden et al. (2017), and close to the canonical TyrXXXLys reductase site (amino acids 186–190). By analogy with the Boyden et al. data, p.Gly182Ser is likely to lead to a structural protein change rather than affect glycosylation or phosphorylation.

### *KDSR* expression and ceramide immunolabeling are reduced in patient skin

Quantitative PCR was performed using whole skin RNA from patient 1, patient 2, and four healthy individuals (data shown in Supplementary Figure S4 online; probe details in Supplementary Table S2 online). *KDSR* expression was found to be reduced in both affected individuals, but not dramatically (70–80% of control; see Supplementary Figure S4a). Expressions of *FLG*, *CERS3*, *IVL*, *KRT10,* and *KRT14* were increased in both patients (see Supplementary Figure S4b–f). Immunofluorescence staining was performed on skin sections from patient 1, patient 2, and a control individual to examine changes in protein levels or localization. KDSR labeling was not visibly reduced in patient skin (see Supplementary Figure S5 online; antibody details in Supplementary Table S3 online). Staining with an anti-ceramide antibody showed reduced (but not absent) ceramide levels in patient skin, supporting the hypothesis that *KDSR* mutations lead to dysregulation of ceramide biosynthesis, although the broad reactivity of the antibody (which recognizes ceramide-2, ceramide-3, ceramide-5, ceramide C14, ceramide C16, and dihydroceramide C16, but not sphingosine or DHS) limits further interpretation. In keeping with the gene expression changes observed, immunoreactivity of CERS3, filaggrin, and loricrin was increased in both patients (see Supplementary Figure S5). Taken together, these alterations suggest that reduction of KDSR activity leads to diminished levels of ceramide in skin with increased or precocious expression of terminal differentiation markers such as keratin 10, involucrin, filaggrin and loricrin.

### *KDSR* mutations lead to variable alterations in skin ceramides

**Figure 4:**
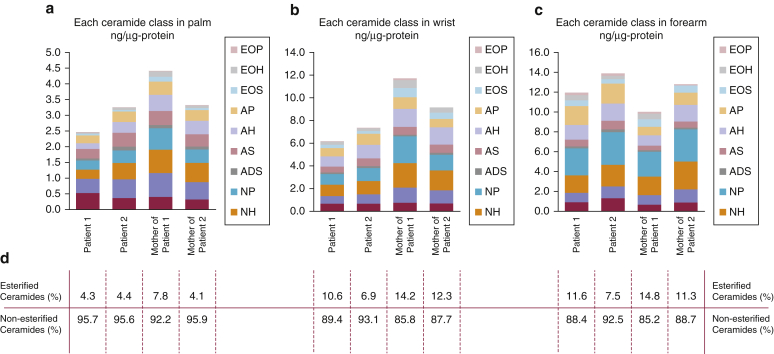
**Tape stripping and liquid chromatography-mass spectrometry analysis shows reduced percentages of esterified ceramides in patient 1 and patient 2 skin.** (**a**) In palm skin, total ceramide levels in patient 1 are reduced compared with his mother, although differences are not observed in patient 2, who had a milder clinical phenotype. (**b**) Total ceramide levels are also reduced in wrist skin in both patients with respect to their mothers, more so for patient 1. (**c**) In forearm skin (clinically normal), total ceramide levels are not reduced in patient skin. (**d**) Subanalysis of the relative percentages of esterified and non-esterified ceramides shows reduced levels of esterified ceramides for all patient samples compared with those of their respective mothers, with the exception of the palm sample from the mother of patient 2. A more detailed analysis of these data are presented in Supplementary Tables S4 and S5.

The levels of 11 major ceramide species in the skin of the forearm, wrist, and palm were assessed by tape stripping and liquid chromatography-mass spectrometry analysis (Figure 4, and Supplementary Tables S4 and S5 online for full details). In the forearms of patients 1 and 2 (uninvolved skin), there was no significant difference in the total ceramide, ceramide components, or average carbon numbers between the affected individuals and their unaffected mothers (see Supplementary Table S1). In contrast, in the affected wrist skin, the levels of total ceramide, CER[EOS], CER[EOH], CER[NP], CER[NH], and CER[NS], were reduced in the patients’ samples. Additionally, the average carbon numbers of ceramides indicated that short chain ceramides, CER[NDS], CER[NS], and CER[AS], were relatively increased in the patients’ skin. However, because of the small number of samples, statistical analyses could not be performed. Likewise, in the affected palm skin samples, the level of total ceramide was decreased in patient 1 compared with his mother. In contrast, there was no difference in the levels of total or individual ceramides between patient 2 and his mother. This discrepancy may be explained by the milder phenotype in patient 2 compared with patient 1. The average carbon numbers of ceramides showed that short chain ceramides, CER[NDS], CER[NS], CER[NP], CER[ADS], CER[AS], and CER[AP], were relatively increased in both patients’ palms. There was a relative (but not absolute) increase of short chain ceramides in wrist and palm. Because total ceramide levels decrease, it is more likely that overall there is a decrease in longer ceramides. KDSR is one of the key enzymes involved in the de novo pathway of sphingolipid synthesis, acting between serine palmitoyl transferase and ceramide synthase. Therefore, KDSR deficiency may affect this cascade and lead to a reduction in the levels of synthesis of total and downstream products.

### *KDSR* mutations reduce platelet number and function

**Figure 5:**
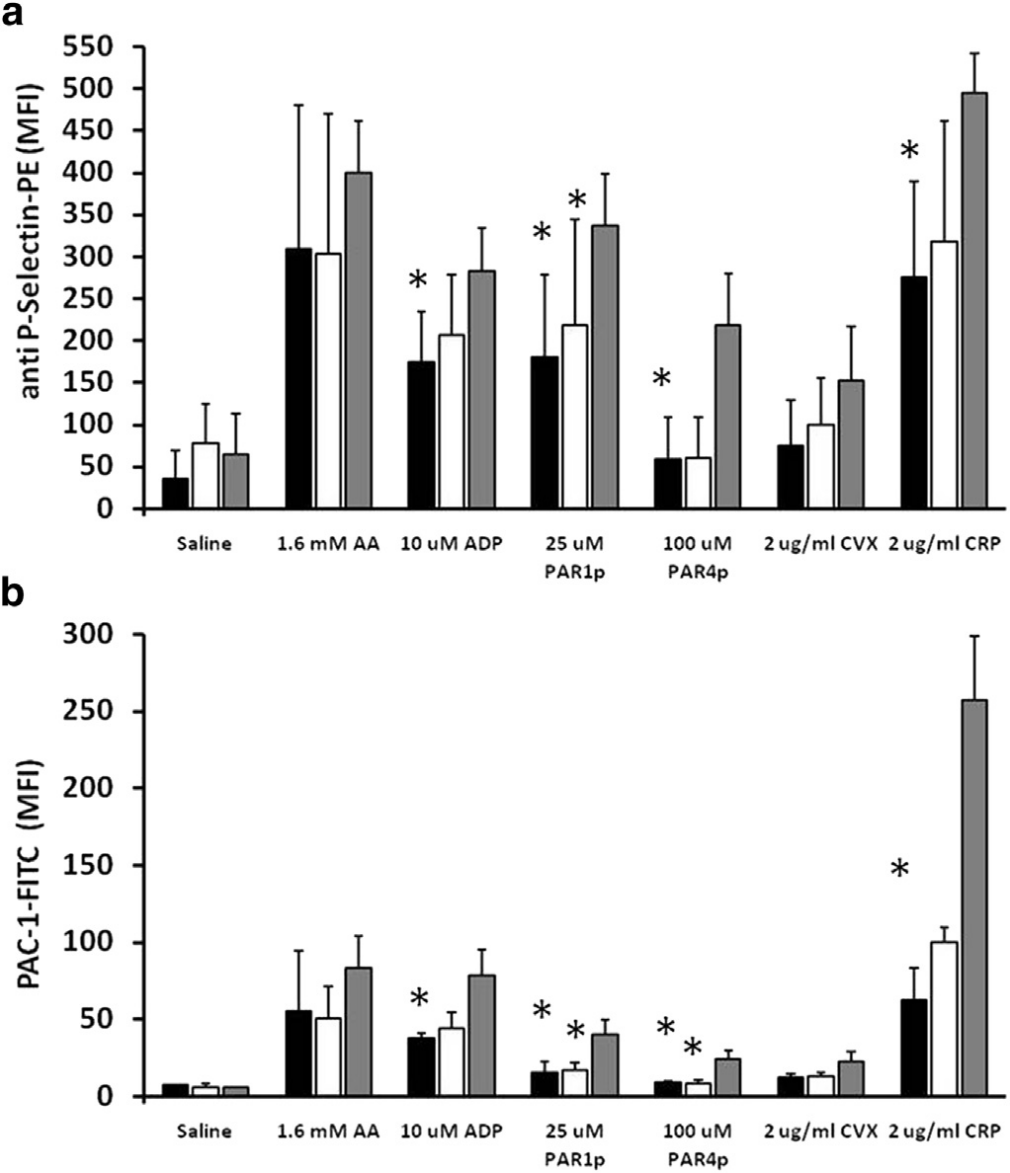
**Functional differences are present in platelets from patients 1 and 2 compared with control samples.** Platelets from compound heterozygotes of the mutations c.413T>G and c.417+3A>C in *KDSR*, their heterozygous mothers (carrying the c.417+3A>C mutation), and healthy unrelated control subjects (combined data from two subjects) were stimulated under static conditions (30 minutes at room temperature) with agonist (1.6 mmol/L arachidonic acid [AA], 10 μmol/L adenosine diphosphate [ADP], 25 μmol/L PAR1 peptide [PAR1p], 100 μmol/L PAR4 peptide [PAR4p], 2 μg/ml convulxin [CVX], and 2 μg/ml collagen-related peptide [CRP]) in the presence of both PAC-1-FITC and anti-CD62P-PE monoclonal antibodies. The samples were evaluated by flow cytometry, and the MFIs for (**a**) alpha granule release (anti-CD62P-PE) and (**b**) αIIbβ3 integrin activation (anti PAC-1-FITC) are shown. Values presented are the mean of MFI ± standard error of mean in duplicate samples from the two compound heterozygote patients (black bars), their mothers (white bars), and two parallel controls (gray bars). ^∗^Significant differences (*P* < 0.05, Mann-Whitney test) compared with control samples. M, mol/L; MFI, median fluorescence intensity.

Detailed analysis of platelets was performed in patients 1 and 2, their respective mothers, and healthy control subjects. No morphologic abnormalities in platelets were noted (see Supplementary Figure S6 online), although flow cytometry evaluation of platelet volume was slightly increased in the patients (Table 2). The patients’ platelets expressed normal levels of adhesive surface glycoproteins, but a lower level of phosphatidylserine exposure in terms of basal annexin V binding positive percentages and also reduced thromboplastin expression in unstimulated washed platelets (Table 2). Next, we performed platelet function analysis by evaluating granule release and the conformational change of α_IIb_β_3_ integrin (CD62P and PAC-1, respectively) upon stimulation with different platelet agonists (Figure 5). The increment both in CD62P– and PAC-1–positive platelets was lower in patients than in control subjects, specifically with agonists that are known to activate pathways that are highly dependent on Src family kinases, such as thrombin (PAR1p and PAR4p), collagen (collagen-related peptide), and adenosine diphosphate, but not as evident after arachidonic acid stimulation (Figure 5).

**Table 2:** Blood parameters, platelet size, glycoprotein expression, and annexin V and tissue factor binding in patients 1 and 2, their mothers, and control subjects

	P1	P2	Mother of P1	Mother of P2	Control 1	Control 2
WBC (×10^9^/L)	16.1	8.2	5.2	8.6	7.2	7.8
Hb (g/dl)	13.9	12.7	12.4	13.5	12.8	14.3
Ht (%)	40.2	37.9	36.6	39.9	37.7	42.6
Platelets (×10^9^/L)	24	7	213	226	207	206
FSC (MFI)	32.9	32.9	26.8	27.9	25.7	25.6
CD42b (MFI)	127.2	122.8	152.7	186.2	161.3	199.5
CD42a (MFI)	187.2	189.2	188.1	207.7	180.6	212.8
CD61 (MFI)	209.8	215.3	194.5	237.8	216.7	226.4
CD49b (MFI)	29.5	28.5	33.6	41.1	39.5	31.6
Annexin V (% positive)	2.5	1.7	5.9	4.2	4.7	3.6
Tissue factor (% positive)	4.2	4.4	7.8	5.9	7.5	6.2

Abbreviations: FSC, forward side scatter; Hb, hemoglobin; Ht, hematocrit; MFI, median fluorescence intensity; P1, patient 1; P2, patient 2; WBC, white blood cells.

**Figure 6:**
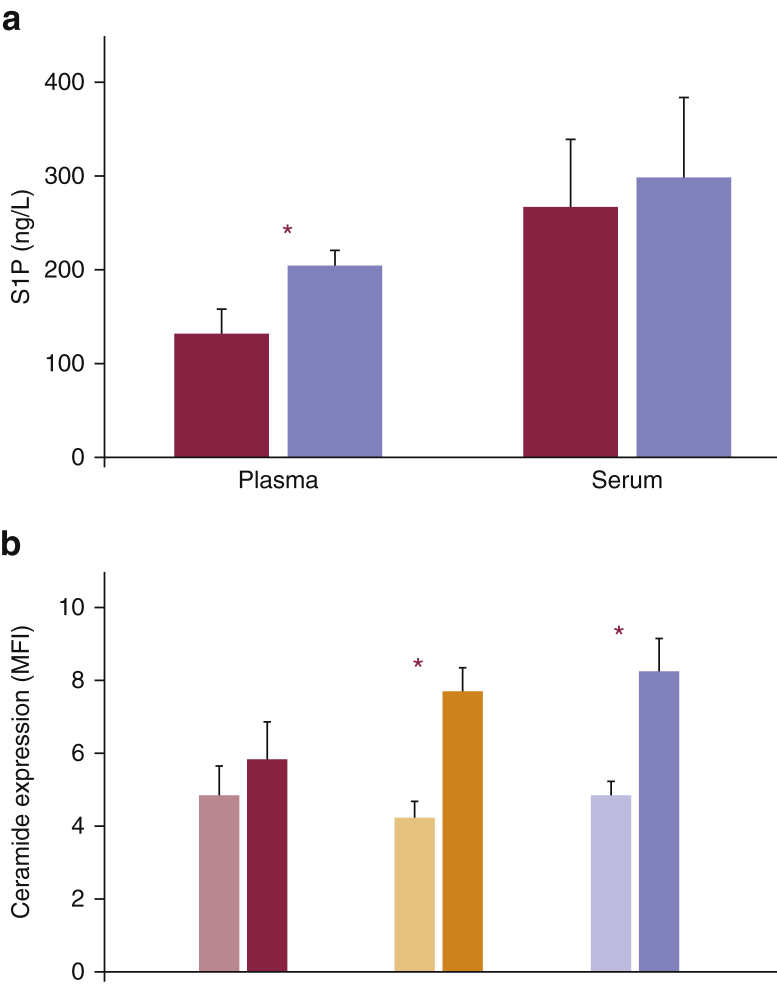
***KDSR* mutations reduce sphingosine 1 phosphate (S1P) and ceramide expression in plasma, serum, and activated platelets.** (**a**) S1P concentration in blood plasma and serum samples obtained from patients 1 and 2 and healthy control subjects. Red bars represent the affected individuals, and violet bars represent two parallel control subjects. (**b**) Ceramide expression in stimulated platelets in response to 250 mmol/L PAR1p. Bars colored in lighter shades of red, orange, and violet represent unstimulated cells, and the bars in darker shades of each respective color indicate activated platelets. The values shown are the means of duplicate samples of (**a**) S1P concentration and (**b**) MFI. The error bars indicate the standard error of the mean. ^∗^Significant differences (*P* < 0.05, Mann-Whitney test) (**a**) in plasma S1P levels between patients and control subjects and (**b**) in ceramide expression between unstimulated and activated platelets. MFI, median fluorescence intensity; PAR1p, PAR1 peptide.

The plasma sphingosine-1-phosphate (S1P) concentration in patient 1, who presented with more severe clinical bleeding, was decreased by 61% compared with control subjects, and the equivalent measure in patient 2 was reduced by 45% (Figure 6a). The observation that serum S1P levels compared with those of control subjects were diminished in both patients by only 45% and 36%, respectively, suggests that erythrocytes contribute to most of the S1P being released in patient samples during blood clotting. Surface-exposed ceramide in human platelets were investigated with an antibody recognizing C24:0 ceramide levels, the predominant form of ceramide present in human platelets (Chen et al., 2013). This antibody detected a significant increase in ceramide levels in the plasma membrane of controls and carriers of the c.417+3A>C mutation after platelet activation, whereas the intensity of immunostaining was not changed significantly in affected patients (Figure 6b).

## Discussion

In this study, we identified biallelic mutations in *KDSR* in patients with defective keratinization and thrombocytopenia, implicating *KDSR* in the pathobiology of hereditary palmoplantar keratodermas and ichthyosis, as recently shown by Boyden et al. (2017), but also showing that KDSR has an important additional role in platelet biology. Our data suggest that mutations in *KDSR* impair ceramide biosynthesis pathways and function in skin and platelets.

Clinically, the skin phenotypes in our patients were diverse: two patients had localized hyperkeratosis/keratoderma, and the other two had generalized harlequin-like ichthyosis. Neither of these forms of abnormal cornification resembled the progressive symmetric erythrokeratoderma reported in association with *KDSR* mutations by Boyden et al. (2017). Collectively, most of the mutations are loss-of-function but, at present, there does not appear to be a basis for clear genotype-phenotype correlation.

Before the discovery of human mutations in *KDSR*, data linking this gene to disease have been very limited, aside from a missense variant in the bovine ortholog of *KDSR* that was proposed to cause spinal muscular atrophy (Krebs et al., 2007). Intriguingly, however, a de novo deletion of human chromosome arm 18q has been reported previously in an infant with lethal harlequin ichthyosis (Stewart et al., 2001): this child’s karyotype was 46,XY,del(18)(q21.3). The authors hypothesized that the causative gene may be located at or distal to 18q21.3 and that this deletion may have unveiled this autosomal recessive disorder. Indeed, *KDSR* is located at 18q21.33, and thus we speculate that loss of *KDSR* may have been responsible for this individual’s phenotype. The vast majority of cases of harlequin ichthyosis have biallelic mutations in the lipid transporter gene *ABCA12* (Akiyama, 2014), but this previous report (Stewart et al., 2001) and our current findings in two further subjects with harlequin ichthyosis identify *KDSR* as a possible additional candidate gene for non-*ABCA12* harlequin ichthyosis.

The mutations we identified in *KDSR* are predominantly loss of function, leading to reduced ceramide synthesis with a relative reduction of esterified ceramides evident in our tape stripping and liquid chromatography-mass spectrometry analyses. Ceramides are a class of sphingolipids, a family of lipids present in eukaryotes, which are involved in a variety of key physiologic functions in the skin, brain, immune system, and blood vessels (Wegner et al., 2016). Ceramides are vital not only for membrane structure integrity but are also essential for critical signaling processes such as cell cycle arrest, migration, chemotaxis, adhesion, and differentiation (Wegner et al., 2016). Additionally, ceramides are relevant to proliferation, inflammation, apoptosis, and autophagy in the context of stress (Uchida, 2014). There are more than 1,000 ceramide species, most of which are present in skin stratum corneum (Kihara, 2016). The major route of ceramide formation is the salvage pathway, which delivers 50–90% of the ceramide and uses hydrolysis of sphingomyelin by sphingomyelinase (Linn et al., 2001). Ceramide can be also be synthesized de novo in the endoplasmic reticulum (Linn et al., 2001). The first step in the de novo pathway of ceramide synthesis is catalyzed by serine palmitoyl transferase, condensing l-serine and a fatty acid to generate 3-KDS. Subsequently, reduction of 3-KDS by KDSR produces DHS. DHS is the substrate of ceramide synthases, a group of six enzymes, which bind fatty acids of varying lengths to the amide group of DHS, thus giving rise to a variety of dihydroceramides (Levy and Futerman, 2010). Finally, dihydroceramide desaturase creates a double bond between positions 4 and 5, generating ceramide.

Patients with mutations in *KDSR* also exhibit progressive thrombocytopenia and a moderate functional platelet defect that develops early in life. The most likely explanation for the reduction in platelet count is diminished S1P synthesis. With relevance to onset of platelet loss, transplacental sphingosine stored in erythrocytes during fetal life may account for thrombocytopenia not being evident at birth. Instead, this phenomenon may manifest once the release from these cells, with a life span of 4 months, is compromised. In thrombopoiesis, both extracellular and intracellular normal levels of this lipid mediator are essential in pro-platelet shedding from megakaryocytes in genetically deficient mice (Zhang et al., 2012, Zhang et al., 2013). Therefore, defects in platelet formation and release in the final stage of thrombopoiesis may contribute to the pathogenesis of thrombocytopenia in *KDSR* patients. Moreover, the functional defects associated with mutations in *KDSR* could be related to the reduced synthesis of not only S1P but also ceramide. Previous studies in knockout mice have shown that platelets defective in S1P or ceramide fail to activate normally and that exogenous ceramide or S1P is able to rescue the phenotype of defective platelet secretion and aggregation (Munzer et al., 2014, Urtz et al., 2015).

The platelet abnormalities in patients proved difficult to treat with conventional approaches, but an alternative strategy might be to use drugs such as fingolimod and related S1P receptor-targeting drugs that act as agonists upon initial binding to the S1P receptor. Fingolimod administration causes a rapid increase in platelet numbers in mice (Zhang et al., 2012), suggesting acute agonistic action of the drug on megakaryocyte S1P receptor-induced platelet release. Thus, it could be possible, in patients with reduced but not absent KDSR enzymatic activity, to therapeutically regulate platelet deficiencies by targeting the S1P receptor. Regarding treatment of the skin, we saw no or limited response to systemic retinoid (acitretin) in three subjects (patients 1, 2, and 3). In contrast, use of isotretinoin in individuals with *KDSR* mutations and a progressive symmetric erythrokeratoderma phenotype was reported to be very effective (Boyden et al., 2017). Between our study (patient 3) and that of Boyden et al. (2017), there were two patients who died in early infancy. Although there are currently no data specifically implicating *KDSR* mutations as being any more likely to lead to increased infant mortality over other forms of congenital ichthyosis, this potentially poor outcome will need to be reviewed as more cases of *KDSR* mutations are documented.

In conclusion, our data add to recent findings by Boyden et al. (2017) in showing that defective ceramide biosynthesis due to mutations in *KDSR* is responsible for some forms of local hyperkeratosis and generalized ichthyosis. Moreover, we show that the *KDSR* mutations we identified are also associated with accompanying thrombocytopenia. Our work therefore extends knowledge about ceramides in skin disease and provides original insights into ceramides and platelet biology, with collective implications for patient diagnostics, prognostics, and therapeutics.

## Materials and Methods

The full description of all materials and methods used in this study for venous blood sampling for DNA, platelet, plasma, and serum studies, as well as methodology for whole-exome sequencing (including reads and coverage), cell culture and transfection, immunofluorescence microscopy, quantitative PCR, and platelet microscopy and flow cytometry are provided in the Supplementary Materials online.

### Yeast strain and medium

The yeast *Saccharomyces cerevisiae* strain KHY625 (*MATa* *ura3 his3 trp1 leu2* Δ*tsc10::LEU2*; Kihara and Igarashi, 2004) harboring a *URA3* marker-containing plasmid was grown on synthetic complete minus uracil (0.67% yeast nitrogen base, 2% d-glucose, 0.5% casamino acids, 20 mg/L adenine, and 20 mg/L tryptophan) plates with or without 5 μmol/L phytosphingosine and 0.0015% Nonidet P-400 (dispersant) at 30 °C.

### Plasmid generation

Human *FVT-1*/*KDSR* cDNA was digested from the pAK591 plasmid (Kihara and Igarashi, 2004) and cloned into pCE-puro 3xFLAG-1, the mammalian expression vector designed for N-terminal 3xFLAG-tagged protein production. Four of the identified mutations (F138C, Δ5, Δ5Δ6, and Q271E) were created using the QuikChange Site-Directed Mutagenesis Kit (Agilent Technologies, Santa Clara, CA), and the primers listed in Supplementary Table S1. The E75Nfs*2 mutant was produced by amplifying the mutated *KDSR* gene using the primers KDSR-F and KDSR E75Nfs*2, respectively (see Supplementary Table S1), followed by cloning into the pCE-puro 3xFLAG-1 vector. For expression in yeast, wild-type and mutant *KDSR* plasmids were transferred into pAKNF316 (*CEN*, *URA3* marker), the yeast expression vector designed to produce N-terminally 3xFLAG-tagged protein under the control of a glyceraldehyde 3-phosphate dehydrogenase (*GAPDH*) promoter.

### Immunoblotting

Immunoblotting was performed as described previously (Kitamura et al., 2015) using anti-FLAG M2 antibody (1.85 μg/ml; Sigma, St. Louis, MO) as the primary antibody and an horseradish peroxidase-conjugated anti-mouse IgG F(ab′)_2_ fragment (diluted 1:7,500; GE Healthcare Life Sciences, Piscataway, NJ) as the secondary antibody.

### In vitro 3-KDS reductase assay

Cells were suspended in buffer A (50 mmol/L Tris-HCl [pH 7.5], 10% glycerol, 150 mmol/L NaCl, 1 mmol/L EDTA, 1× protease inhibitor mixture [Complete EDTA free; Roche Diagnostics, Basel, Switzerland], 1 mmol/L phenylmethylsulfonyl fluoride, and 1 mmol/L dithiothreitol]) and lysed by sonication. After ultracentrifugation (100,000*g*, 30 minutes, 4 °C), the pellet was suspended in buffer A and was used as the total membrane fraction. Protein amounts were quantified using the Pierce BCA Protein Assay Kit (Thermo Fisher Scientific, Waltham, MA). In vitro KDS reductase assay was performed by incubating the total membrane fraction (1 μg) with 1 mmol/L nicotinamide adenine dinucleotide phosphate and 10 μmol/L KDS (C18, Matreya, State College, PA) at 37 °C for 1 hour. Lipids were extracted by mixing with successive additions of 3.75 volume of chloroform/methanol/HCl (100:200:1, volume/volume/volume), 1.25 volume of chloroform, and 1.25 volume of water. Phases were separated by centrifugation (20,000*g*, room temperature, 3 minutes). The resulting organic (lower) phase was recovered, dried, and dissolved in methanol. The reaction product DHS was detected by ultra performance liquid chromatography coupled with electrospray ionization tandem triple quadrupole MS (Xevo TQ-S; Waters, Milford, MA). The ultra performance liquid chromatography solvent systems and electrospray ionization condition were described previously (Yamamoto et al., 2016). DHS was detected by multiple reaction monitoring by selecting the *m/z* value of 302.2 at Q1 and the *m/z* value of 266.0 at Q3 with the collision energy setting at 20 V in positive ion mode (see Supplementary Table S5). DHS levels were quantified using a standard curve plotted from serial dilutions of DHS (Avanti Polar Lipids, Alabaster, AL) standard. Data were analyzed using MassLynx software (Waters).

### Tape stripping for ceramide analysis

To examine the ceramide species present in the stratum corneum, tape stripping was performed by pressing an acryl film tape (456#40; Teraoka Seisakusho, Tokyo, Japan) to the skin of the forearm, wrist, and palm. Five strips measuring 25 mm × 50 mm each were obtained from a single individual. The samples were then subjected to liquid chromatography-mass spectrometry analysis to assess the levels of 11 major ceramide species (Ishikawa et al., 2013, Ohno et al., 2015). The strips were cut into two half-strips, one for lipid analysis and the other for protein analysis. The lipids within the first half-strip were dissolved in 2 ml of chloroform/methanol/2-propanol (10:45:45, volume/volume/volume). N-heptadecanoyl-d-erythro-sphingosine (d18:1/17:0) (Avanti Polar Lipids) was added as an internal control, and its final concentration was 50 nmol/L. This lipid solution was subjected to reversed-phase liquid chromatography/mass spectrometry. The system was an Agilent 1100 Series LC/MSD SL system equipped with a multi-ion source, ChemStation software, a 1,100-well plate auto-sampler (Agilent Technologies) and an L-column octadecylsilyl (2.1 mm inside diameter × 150 mm; Chemicals Evaluation and Research Institute). Chromatographic separation of the lipids was achieved at a flow rate of 0.2 ml/minute using a mobile phase of binary gradient solvent system. Each ceramide species was detected by selected ion monitoring of m/z [M+CH3COO]^−^. Soluble proteins were extracted from the other half-strip with a 0.1-mol/L NaOH of 1% sodium dodecyl sulfate aqueous solution at 60 °C for 150 minutes. The extract solutions were then neutralized with an HCl aqueous solution. After that, soluble proteins were measured using a BCA protein assay kit (Thermo Fisher Scientific, Waltham, MA). Samples were taken from two unaffected mothers (families 1 and 2) as a control.

## ORCIDs

David P Kelsell: http://orcid.org/0000-0002-9910-7144

John A. McGrath: http://orcid.org/0000-0002-3708-9964

WH Irwin McLean: http://orcid.org/0000-0001-5539-5757

## Conflict of Interest

The authors state no conflict of interest.

## References

[bib1] Akiyama M. (2014). The roles of ABCA12 in epidermal lipid barrier formation and keratinocyte differentiation. Biochim Biophys Acta.

[bib2] Aldahmesh M.A., Mohamed J.Y., Alkuraya H.S., Verma I.C., Puri R.D., Alaiya A.A. (2011). Recessive mutations in ELOVL4 cause ichthyosis, intellectual disability, and spastic quadriplegia. Am J Hum Genet.

[bib3] Boyden L.M., Vincent N.G., Zhou J., Hu R., Craiglow B.G., Bayliss S.J. (2017). Mutations in KDSR cause recessive progressive symmetric erythrokeratoderma. Am J Hum Genet.

[bib4] Chen W.F., Lee J.J., Chang C.C., Lin K.H., Wang S.H., Sheu J.R. (2013). Platelet protease-activated receptor (PAR)4, but not PAR1, associated with neutral sphingomyelinase responsible for thrombin-stimulated ceramide-NF-kappaB signaling in human platelets. Haematologica.

[bib5] Eckl K.M., Tidhar R., Thiele H., Oji V., Hausser I., Brodesser S., Preil M.K. (2013). Impaired ceramide synthesis causes autosomal recessive congenital ichthyosis and reveals the importance of ceramide acyl chain length. J Invest Dermatol.

[bib6] Fischer J. (2009). Autosomal recessive congenital ichthyosis. J Invest Dermatol.

[bib7] Ishikawa J., Shimotoyodome Y., Ito S., Miyauchi Y., Fujimura T., Kitahara T. (2013). Variations in the ceramide profile in different seasons and regions of the body contribute to stratum corneum functions. Arch Dermatol Res.

[bib8] Kihara A. (2016). Synthesis and degradation pathways, functions, and pathology of ceramides and epidermal acylceramides. Prog Lipid Res.

[bib9] Kihara A., Igarashi Y. (2004). FVT-1 is a mammalian 3-ketodihydrosphingosine reductase with an active site that faces the cytosolic side of the endoplasmic reticulum membrane. J Biol Chem.

[bib10] Kitamura T., Takagi S., Naganuma T., Kihara A. (2015). Mouse aldehyde dehydrogenase ALDH3B2 is localized to lipid droplets via two C-terminal tryptophan residues and lipid modification. Biochem J.

[bib11] Krebs S., Medugorac I., Rother S., Strasser K., Forster M. (2007). A missense mutation in the 3-ketodihydrosphingosine reductase FVT1 as candidate causal mutation for bovine spinal muscular atrophy. Proc Natl Acad Sci USA.

[bib12] Levy M., Futerman A.H. (2010). Mammalian ceramide synthases. IUBMB Life.

[bib13] Linn S.C., Kim H.S., Keane E.M., Andras L.M., Wang E., Merrill A.H. (2001). Regulation of de novo sphingolipid biosynthesis and the toxic consequences of its disruption. Biochem Soc Trans.

[bib14] Munzer P., Borst O., Walker B., Schmid E., Feijge M.A., Cosemans J.M. (2014). Acid sphingomyelinase regulates platelet cell membrane scrambling, secretion, and thrombus formation. Arterioscler Thromb Vasc Biol.

[bib15] Ohno Y., Nakamichi S., Ohkuni A., Kamiyama N., Naoe A., Tsujimura H. (2015). Essential role of the cytochrome P450 CYP4F22 in the production of acylceramide, the key lipid for skin permeability barrier formation. Proc Natl Acad Sci USA.

[bib16] Oji V., Tadini G., Akiyama M., Blanchet Bardon C., Bodemer C., Bourrat E. (2010). Revised nomenclature and classification of inherited ichthyoses: results of the First Ichthyosis Consensus Conference in Soreze 2009. J Am Acad Dermatol.

[bib17] Radner F.P., Marrakchi S., Kirchmeier P., Kim G.J., Ribierre F., Kamoun B. (2013). Mutations in CERS3 cause autosomal recessive congenital ichthyosis in humans. PLoS Genet.

[bib18] Sakiyama T., Kubo A. (2016). Hereditary palmoplantar keratoderma “clinical and genetic differential diagnosis”. J Dermatol.

[bib19] Stewart H., Smith P.T., Gaunt L., Moore L., Tarpey P., Andrew S. (2001). De novo deletion of chromosome 18q in a baby with harlequin ichthyosis. Am J Med Genet.

[bib20] Uchida Y. (2014). Ceramide signaling in mammalian epidermis. Biochim Biophys Acta.

[bib21] Urtz N., Gaertner F., von Bruehl M.L., Chandraratne S., Rahimi F., Zhang L. (2015). Sphingosine 1-phosphate produced by sphingosine kinase 2 intrinsically controls platelet aggregation in vitro and in vivo. Circ Res.

[bib22] Wegner M.S., Schiffmann S., Parnham M.J., Geisslinger G., Grosch S. (2016). The enigma of ceramide synthase regulation in mammalian cells. Prog Lipid Res.

[bib23] Xiong H.Y., Alipanahi B., Lee L.J., Bretschneider H., Merico D., Yuen R.K. (2015). RNA splicing. The human splicing code reveals new insights into the genetic determinants of disease. Science.

[bib24] Yamamoto S., Yako Y., Fujioka Y., Kajita M., Kameyama T., Kon S. (2016). A role of the sphingosine-1-phosphate (S1P)-S1P receptor 2 pathway in epithelial defense against cancer (EDAC). Mol Biol Cell.

[bib25] Zhang L., Orban M., Lorenz M., Barocke V., Braun D., Urtz N. (2012). A novel role of sphingosine 1-phosphate receptor S1pr1 in mouse thrombopoiesis. J Exp Med.

[bib26] Zhang L., Urtz N., Gaertner F., Legate K.R., Petzold T., Lorenz M. (2013). Sphingosine kinase 2 (Sphk2) regulates platelet biogenesis by providing intracellular sphingosine 1-phosphate (S1P). Blood.

