# Supplementary material for: Biallelic Mutations in KDSR Disrupt Ceramide Synthesis and Result in a Spectrum of Keratinization Disorders Associated with Thrombocytopenia
Source: J Invest Dermatol. 2017 Nov;137(11):2344–53. doi: 10.1016/j.jid.2017.06.028 (PMC5646945; doi:10.1016/j.jid.2017.06.028)
Supplement: Supplementary Data [file mmc1.pdf]

Supplementary material

Materials and methods

Ethics statement

This study was conducted in compliance with the Declaration of Helsinki and all participants provided informed consent. This study was also approved by the relevant institutional authorities, namely the St Thomas' Hospital Ethics Committee, UK ("Molecular basis of inherited skin diseases" 07/H0802/104), the Bioethics Committee of the Nagoya University Graduate School of Medicine, Japan, and the Ethics Committee of Hospital Universitario Reina Sofía, Murcia, Spain.

Blood sampling

Venous blood was drawn from affected individuals, their mothers, and two parallel healthy controls into 7.5% K3 EDTA tubes (for blood counts and DNA isolation), buffered 0.105 M sodium citrate (for platelet function studies), or in empty tubes (for preparation of serum) using a 20-gauge needle. Samples were maintained at room temperature until processing. Platelet-rich plasma (PRP) and platelet-poor plasma (PPP) were separated from blood samples by stepwise centrifugations at 140 x g for 10 min and then 1200 x g for 20 min at room temperature (RT). Serum was obtained from non-anticoagulated blood by incubation at 37°C for 30 min followed by centrifugation (1200 x g, 20 min). PPP and serum aliquots were stored frozen at -80°C until used in the human sphingosine 1 phosphate enzyme-linked

immunosorbent assay (Shanghai Crystal Day Biotech Co., LTD, Shanghai, China). For some studies, washed platelets resuspended in Tyrode´s buffer (Guerrero et al., 2005) were used.

Whole-exome sequencing

Genomic DNA was extracted and whole-exome capture was performed by in-solution hybridization (Agilent All Exon V4 kit, Agilent Technologies, Santa Clara, CA, USA). Massively parallel sequencing was performed with the Illumina HiSeq 2000 platform with 100-bp paired-end reads (San Diego, CA). The reads produced were aligned to the reference human genome using the NovoAlign software package (Novocraft Technologies Sdn Bhd, Selangor, Malaysia). PCR duplicates were removed using MarkDuplicates in Picard tools (https://broadinstitute.github.io/picard/). Candidate variants were called using VarScan2 (http://massgenomics.org/varscan) and annotated using ANNOVAR (http://annovar.openbioinformatics.org/). Common variants defined by >1% minor allele frequency in ExAC (http://exac.broadinstitute.org/), 1000 genomes (http://www.1000genomes.org/), or ESP6500 (http://evs.gs.washington.edu/EVS/) were excluded from analysis.

- Family 1: Mean exome coverage was 42.96. 10x and 20x coverage were 92.25% and 79.11% respectively.
- Family 2: Mean exome coverage was 104.16. 10x and 20x coverage were 97.31% and 94.51% respectively.
- Family 3: Mean exome coverage was 129.83. 10x and 20x coverage were 95.49% and 91.89% respectively.

- Family 4: Mean exome coverage was 78.5. 10x and 20x coverage were 98.0% and 94.7%, respectively.

Cell culture and transfection

HEK 293T cells were grown in Dulbecco's Modified Eagle's Medium (D6429; Sigma, St. Louis, MO) supplemented with 10% FBS, 100 U/mL penicillin, and 100 µg/mL streptomycin in a 5% CO_2 incubator at 37 °C. Dishes were pre-coated with 0.1 mg/mL collagen (Cellmatrix type I-P, Nitta Gelatin, Osaka, Japan). Transfections were conducted using Lipofectamine Plus™ Reagent (Thermo Fisher Scientific, Waltham, MA), according to the manufacturer's instructions.

Immunofluorescence microscopy

Following informed consent, skin samples were taken from patients and healthy controls under local anesthetic. Samples were placed in Michel's medium at room temperature during transportation. Skin samples were washed in 0.1 M Dulbecco's Phosphate Buffered Saline (PBS) for 1 hour at 4 °C, mounted in Optimal Cutting Temperature compound (Agar Scientific, Stansted, UK) and immediately frozen in liquid nitrogen cooled n-heptane. Cryostat sections of 5 µm were cut and transferred onto Superfrost™ Plus slides (Thermo Scientific, UK). The samples were air dried and stored in a -20 °C freezer until required. Prior to staining, slides were air dried for 10 minutes, immersed in PBS for 5 minutes and incubated with goat/rabbit serum (Sigma-Aldrich) for 2 minutes at room temperature. Primary antibodies were diluted in 1% bovine serum albumin (BSA) (Sigma-Aldrich, Gillingham, Dorset, UK) in PBS at the

desired concentration (see Supplementary Table S3 for the list of primary antibodies used). A negative control was prepared with serum without adding any primary antibody. The slides were then incubated with the primary antibody or negative control solution for 1 hour at 37 °C or overnight at 4 °C in a humidified chamber. They were then washed twice in PBS for 10 minutes and incubated with the relevant fluorescein-conjugated secondary antibodies (Vector Labs, CA) diluted 1:500 in 1% BSA/PBS for 1 hour at room temperature in a dark humidified air chamber. The samples were washed twice in PBS for 10 minutes and then twice in distilled water for 10 minutes before being mounted in glycerol/PBS-containing vector shield with DAPI (Vector Labs). Images of the slides were captured with a Nikon Eclipse E600 epifluorescence microscope fitted with a Jenoptik CF Cool digital camera (Jenoptik, Jena, Germany).

Flow cytometry

Platelet expression of major platelet membrane glycoproteins (GP) (GPIa [CD49b], GPIbα [CD42b], CD42a [GPIX], integrin β3 [CD61]), was assessed by flow cytometry in PRP through a direct standard technique with appropriate labeled monoclonal antibodies (Becton Dickinson, San Jose, CA). For analysis of surface-expressed P-selectin (marker of alpha granule release) and binding of PAC-1 (marker for activated αIIbβ3), diluted PRP (~30x10^9/L platelets) was stimulated under static conditions (30 min at room temperature) with the desired agonist concentration in the presence of both anti-CD62-PE and PAC1-FITC antibodies (Becton Dickinson). Tissue factor expression (binding of anti-CD142 antibody, Becton Dickinson) and detection of phosphatidylserine using fluorescein labelled Annexin V (Becton Dickinson) was analyzed in unstimulated, washed platelets. To analyze for ceramide expression in washed

platelets, cells were incubated with or without 100 µM PAR1 peptide [PAR1p] and an antibody recognizing C-16 and C-24 ceramide (LifeSpan BioScience, Seattle City, WA). Platelets were washed and incubated with a FITC-conjugated anti-mouse IgM (LifeSpan BioScience). Samples were then run in the FACSCalibur flow cytometer (Becton Dickinson) and the percentage or median fluorescence intensity (MFI) or percentage of positively stained cells was analyzed using the CellQuest software (Becton Dickinson).

Electron microscopy analysis of platelet ultrastructure

PRP samples were fixed in 1.25% glutaraldehyde, washed and post fixed in 1% osmic acid containing 1.5% potassium ferrocyanide, dehydrated using graded alcohols and propylene oxide and embedded in Epon as described previously (Navarro-Nunez et al., 2011). Embedded samples were sectioned, stained, and visualized using a Philips Tecnai 12 transmission electron microscope and a Megaview III camera (FEI, Hillsboro, OR).

Quantitative reverse transcription polymerase chain reaction (qPCR)

A 20 µL reaction mixture was made up of 2 µL of cDNA template, 10 µL of Taqman® Mastermix (Thermo Fisher Scientific, UK), 1 µL of gene expression assay (Thermo Fisher Scientific, Paisley, UK) and 7 µL of diethyl pyrocarbonate (DEPC)-treated water (see Supplementary Table S4 for the list of probes used). Each reaction was performed in triplicate to correct for pipetting errors. The mixture was then pipetted into a MicroAmp Optical 96-well plate (Applied

Biosystems) and placed into the ABI 7900HT Fast Real-Time PCR System (Applied Biosystems, Carlsbad, CA). The samples were heated to 50 °C and then 95 °C for 10 minutes for AmpliTaq Gold DNA polymerase activation, followed by 40 cycles at 95 °C for 15 seconds and 60 °C for 1 minute for denaturation, annealing, and elongation. A water sample was included as a no template control to exclude contamination. The mean C_T value for each assay was determined and all experiments were repeated on three independent occasions to enable statistical analyses and correct for experimental viability.

References for Supplementary Methods

Guerrero JA, Lozano ML, Castillo J, Benavente-Garcia O, Vicente V, Rivera J. Flavonoids inhibit platelet function through binding to the thromboxane A2 receptor. J Thromb Haemost 2005;3:369-76.

Navarro-Nunez L, Teruel R, Anton AI, Nurden P, Martinez-Martinez I, Lozano ML, et al. Rare homozygous status of P43 beta1-tubulin polymorphism causes alterations in platelet ultrastructure. Thromb Haemost 2011;105:855-63.

Figures and Tables

Figure S1

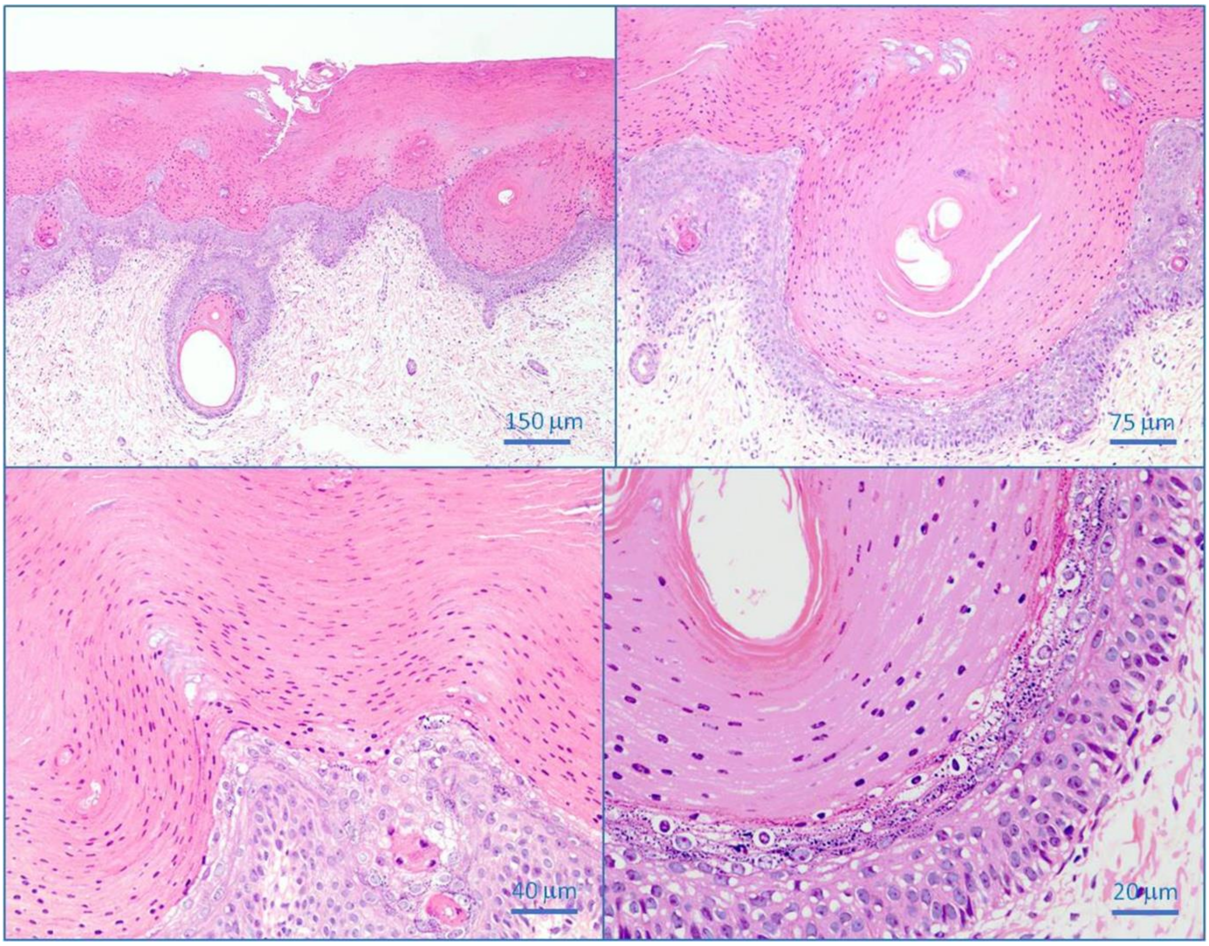

Figure S1. Light microscopic images of a skin biopsy sampled from the upper arm of patient 4. Sections show marked hyperkeratosis with parakeratosis and focal hypergranulosis.

Figure S2

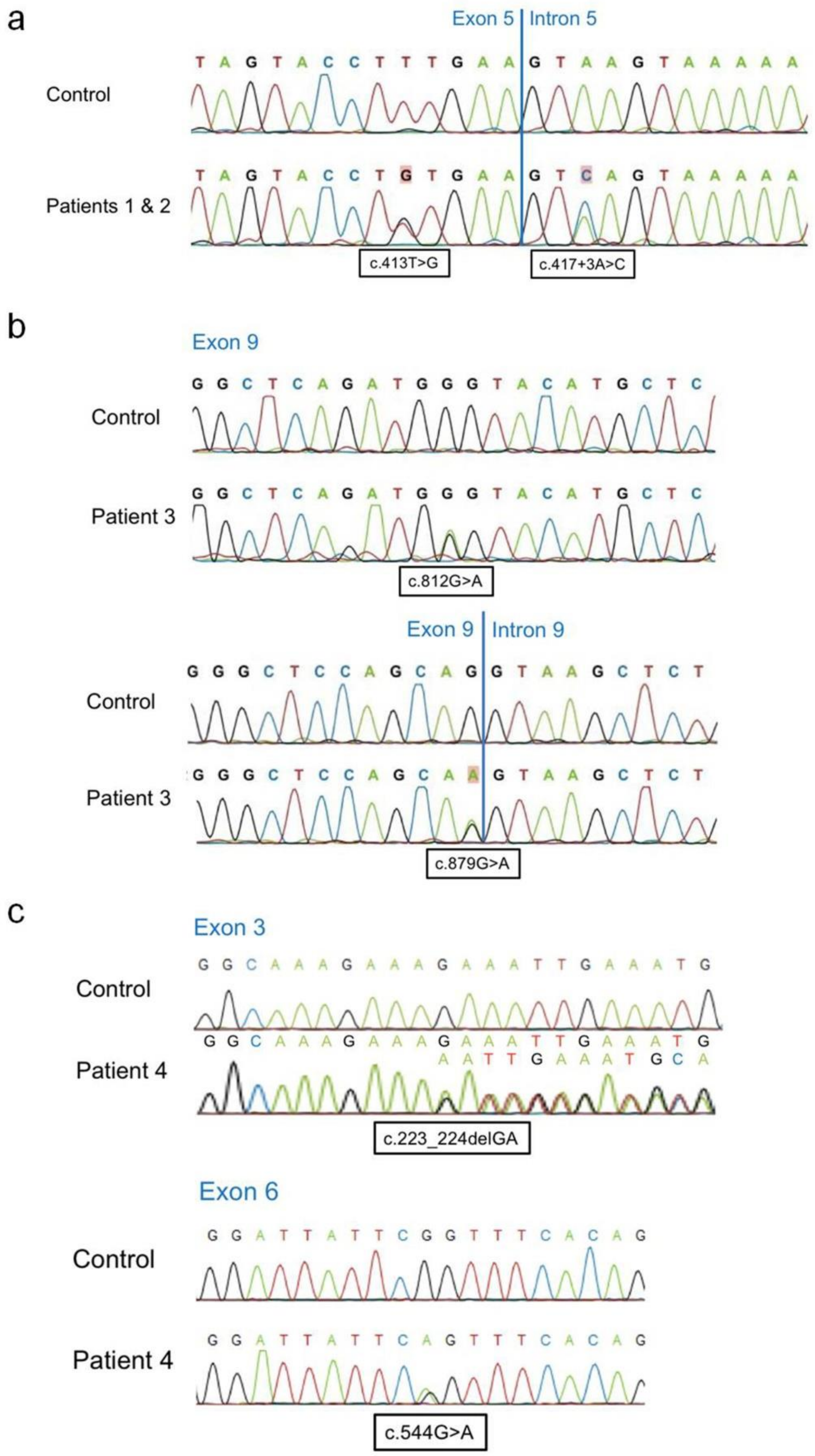

a
Exon 5
Intron 5
T A G T A C C T T T G A A G T A A G T A A A A A
Control
T A G T A C C T G T G A A G T C A G T A A A A A
Patients 1 & 2
c.413T>G
c.417+3A>C
b
Exon 9
G G C T C A G A T G G G T A C A T G C T C
Control
G G C T C A G A T G G G T A C A T G C T C
Patient 3
c.812G>A
Exon 9
Intron 9
G G G C T C C A G C A G G T A A G C T C T
Control
G G G C T C C A G C A A G T A A G C T C T
Patient 3
c.879G>A
c
Exon 3
G G C A A A G A A A G A A A T T G A A A T G
Control
G G C A A A G A A A G A A A T T G A A A T G
A A T T G A A A T G C A
Patient 4
c.223_224delGA
Exon 6
G G A T T A T T C G G T T T C A C A G
Control
G G A T T A T T C A G T T T C A C A G
Patient 4
c.544G>A

Figure S2. Sanger sequencing confirming *KDSR* mutations in genomic DNA. Chromatograms illustrating the six *KDSR* mutations identified in this study. (a) Patients 1 and 2 (from Spain) are both compound heterozygous for the c.413T>G and c.417+3A>C mutations; (b) Patient 3 (from the UK) is compound heterozygous for c.812G>A and c.879G>A; (c) Patient 4 (from Japan) is compound heterozygous for c.223_224delGA and c.544G>A.

Figure S3

a

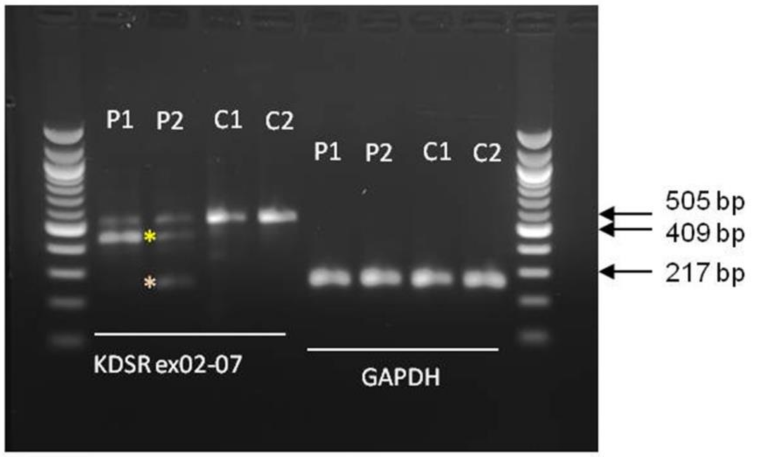

b

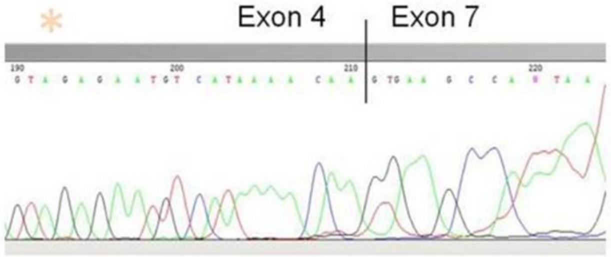

FORWARD PRIMER (within exon 2) 5′-AGGTTCCAGTGGCATCGG-3′
REVERSE PRIMER (within exon 7) 5′-TCTGTGTCTGGTGGGTAAGC-3′

c

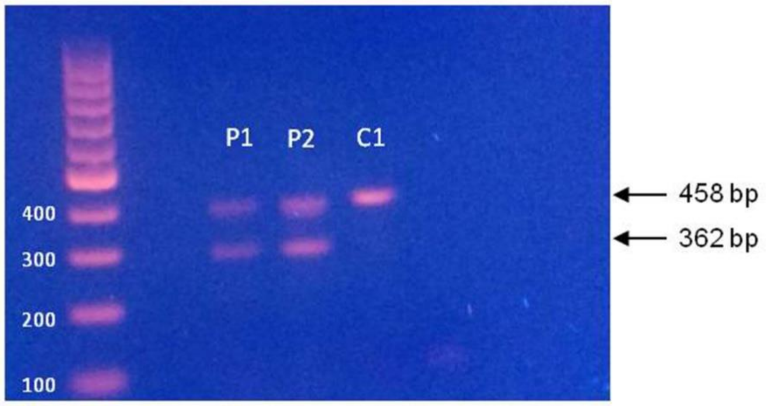

FORWARD PRIMER (within exon 4) 5′-GCATATCAGTTGATGTATCTCAAG-3′
REVERSE PRIMER (within exon 8) 5′- CCATGGACATCTGAATGCAT-3′

Figure S3. The c.417+3A>C mutation in *KDSR* results in aberrant splicing with deletion of either exon 5 or exon 5 and 6. (a) Detection of exon 5 skipping in cDNA prepared from skin samples (Patients 1 and 2; P1 and P2). PCR fragments obtained were separated on a 2% agarose gel. The PCR amplification products of P1 and P2 showed the presence of two or three bands, one with the expected 505 bp product size, plus an additional band (yellow asterisk) with a deleted sequence of ~96 bp corresponding to exon 5, and a further band (pink asterisk) with a deleted sequence of ~288 bp corresponding to exons 5 and 6. The amplification of the control samples (C1 and C2) revealed a single 505 bp band. GAPDH was used as a loading control. The flanking columns show the 100 bp ladder (Biotools, Madrid, Spain); (b) Sanger sequencing of the lower band product (pink asterisk band) reveals adjoined exon 4/7 borders confirming skipping of exons 5 and 6; (c) Detection of exon 5 skipping in cDNA prepared from peripheral blood samples from P1 and P2. PCR fragments obtained were separated on a 2% agarose gel. The PCR amplification products of P1 and P2 showed the presence of two bands, one with the expected 458 bp product size, plus an additional band with a deleted sequence of ~96 bp corresponding to exon 5. The amplification of the control sample (C1) revealed a single 458 bp band. The leftmost column shows the 100 bp ladder (Biotools).

Figure S4

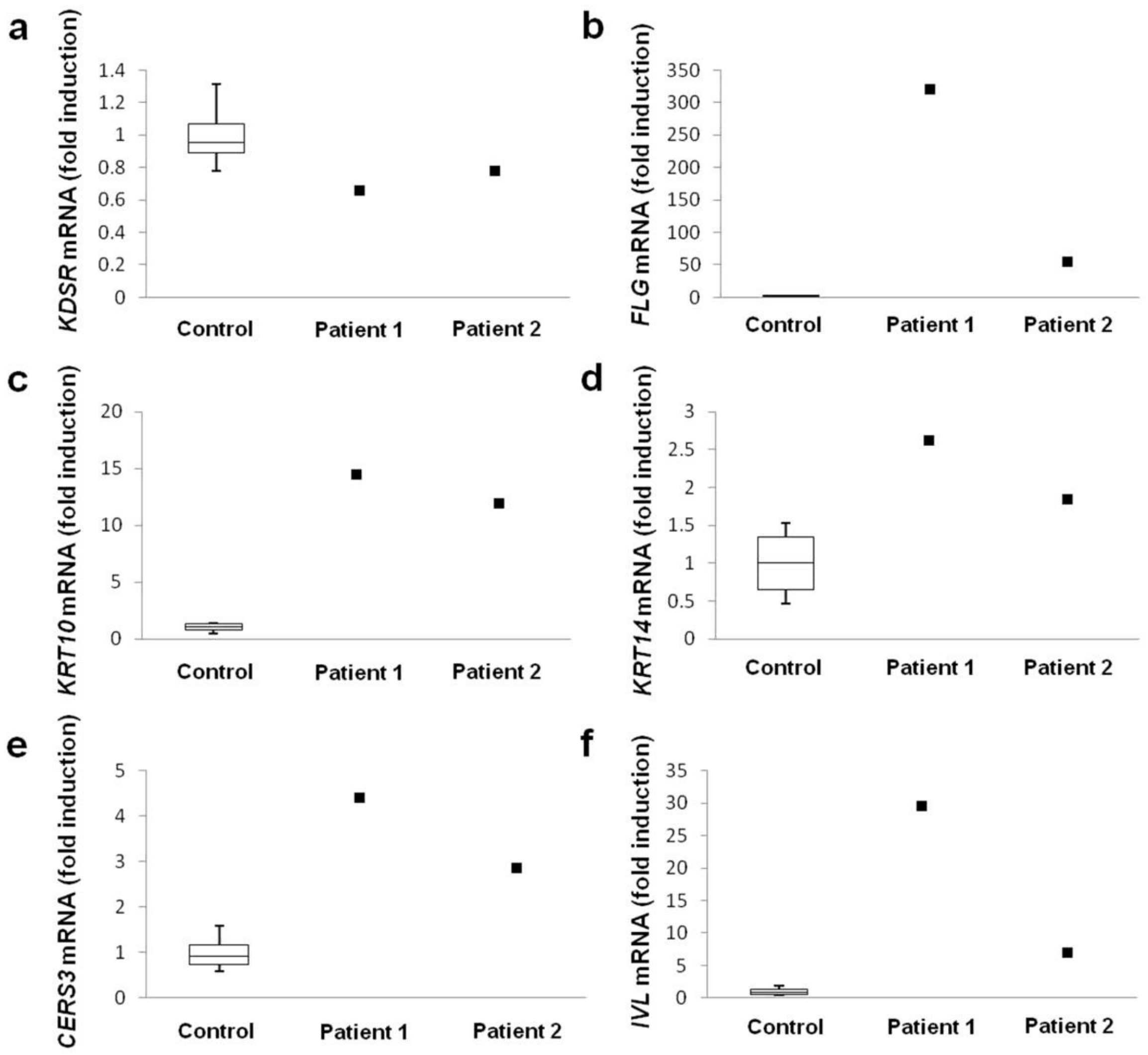

Figure S4. *KDSR* mutations slightly reduce *KDSR* expression but upregulate expression of skin differentiation markers. The mRNA levels of (a) *KDSR*, (b) *FLG*, (c) *KRT10*, (d) *KRT14*, (e) *CERS3*, and (f) *IVL* were evaluated by q-PCR in skin from Patients 1 and 2 and four healthy controls. *18S* was used to normalize gene expression levels.

Figure S5

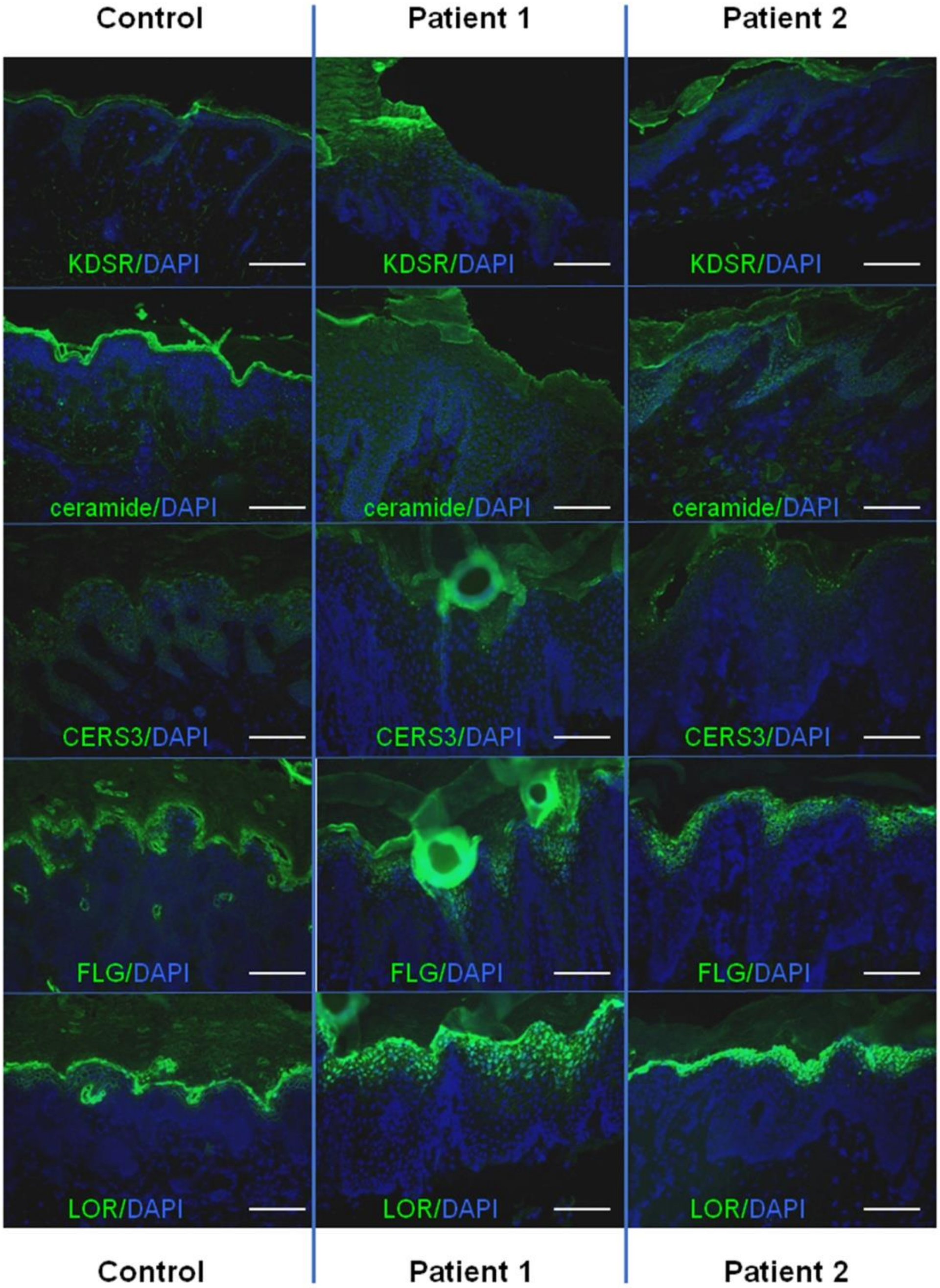

Figure S5. Immunofluorescence analysis in skin from affected patients and control. Immunofluorescence staining reveals reduced ceramide labeling with precocious staining for markers of epidermal differentiation compared to control skin. Bar = 50μm.

Figure S6

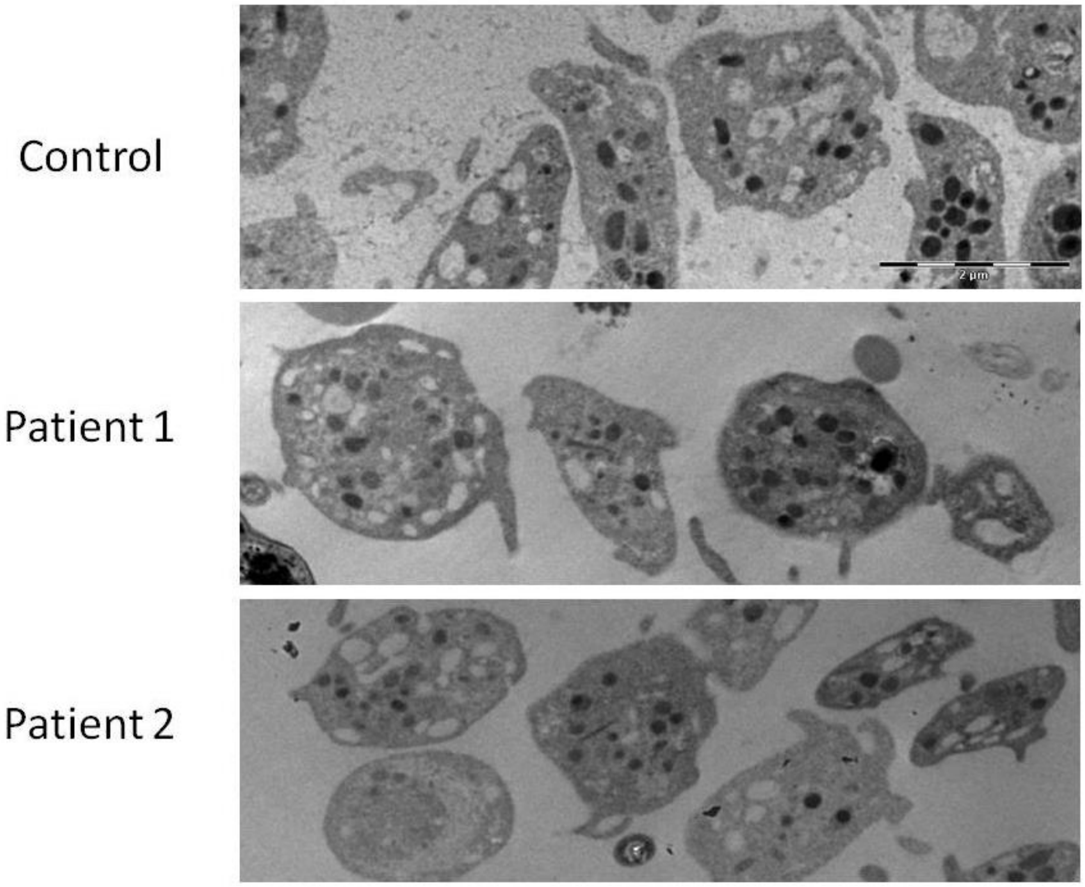

Figure S6. Transmission electron microscopy of platelets shows no morphological differences between patients 1 and 2 and control. Although thrombocytopenia is evident in patient 1 and 2, the ultrastructural appearances of individual platelets show no specific abnormalities.

Supplementary Tables

Primer	DNA sequence
KDSR F138C-F	5’-CTTGAAGTTAGTACCTGTGAAAGGTTAATGAG-3’
KDSR F138C-R	5’-CTCATTAACCTTTCACAGGTACTAACTTCAAG-3’
KDSR Δ5-F	5’-GTAGAGAATGTCATAAAACAAAGGTTAATGAGCATCAATTAC-3’
KDSR Δ5-R	5’-GTAATTGATGCTCATTAACCTTTGTTTTATGACATTCTCTAC-3’
KDSR Δ5Δ6-F	5’-GTAGAGAATGTCATAAAACAAGTGAAGCCATATAATGTCTAC-3’
KDSR Δ5Δ6-R	5’-GTAGACATTATATGGCTTCACTTGTTTTATGACATTCTCTAC-3’
KDSR G271E-F	5’-CCCTTGGCTCAGATGAGTACATGCTCTCGGC-3’
KDSR G271E-R	5’-GCCGAGAGCATGTACTCATCTGAGCCAAGGG-3’
KDSR-F	5’-GGATCCATGCTGCTGCTGGCTGCCGCCTTCC-3’
KDSR Δ10+VSSA-R	5’-CTAGGCAGAGCTTACTTGCTGGAGCCCCTCAGTAATAGAAG-3’
KDSR E75Nfs*2-R	5’-TCAATTTTTCTTTGCCTGCAGCAGC-3’
KDSR G182S-F	5’-CAGTTGGGATTATTCAGTTTCACAGCCTAC-3’
KDSR G182S-R	5’-GTAGGCTGTGAAACTGAATAATCCCAACTG-3’

Table S1. Primer sequences used to generate *KDSR* mutant plasmids for *in vitro* experiments.

Gene	Assay ID
KDSR	Hs00179997_m1
FLG	Hs00856927_g1
CERS3	Hs00698859_m1
IVL	Hs00846307_s1

KRT10	Hs00166289_m1
KRT14	Hs00265033_m1
18S	Hs03003631_g1

Table S2. List of qPCR probes used in this study. All probes were purchased from Thermo Fisher Scientific, Paisley, UK.

Antigen	Product ID	Source
KDSR	bs-13233R	Bioss Inc, Woburn, MA
CERS3	HPA006092	Sigma-Aldrich, St Louis, MO
FLG	SPM181	Abcam, Cambridge, UK
LOR	ab24722	Abcam, Cambridge, UK
Ceramide	MAB_0011	Glycobiotech, Kukels, Germany
DAPI	H-1200	Vector Labs, Burlingame, CA

Table S3. List of primary antibodies used in skin immunofluorescence studies.

Table S4 (see below). Liquid chromatography-mass spectrometry raw data and analysis for tape stripping skin samples.

Table S5 (see below). Additional data for ceramides detected by Liquid chromatography-mass spectrometry including m/z values with corresponding retention times.

Sample		Patient 1			Patient 2			Mother of Patient 1			Mother of Patient 2		
		Palm	Wrist	Forearm	Palm	Wrist	Forearm	Palm	Wrist	Forearm	Palm	Wrist	Forearm
Protein (μg)		64	228	164	61	149	141	84	295	280	146	213	422
Ceramide level (ng/μg-protein)	NDS	0.5	0.7	0.9	0.4	0.6	1.3	0.4	0.7	0.6	0.3	0.7	0.8
	NS	0.5	0.7	0.9	0.6	0.8	1.2	0.8	1.3	1.0	0.6	1.2	1.4
	NH	0.3	1.0	1.7	0.5	1.1	2.2	0.7	2.1	1.9	0.6	1.7	2.8
	NP	0.3	1.0	2.8	0.4	1.2	3.3	0.7	2.4	2.5	0.4	1.4	3.2
	ADS	0.1	0.1	0.2	0.1	0.2	0.3	0.1	0.2	0.1	0.1	0.1	0.2
	AS	0.3	0.5	0.6	0.4	0.7	0.8	0.4	0.7	0.4	0.4	0.7	0.6
	AH	0.2	0.9	1.5	0.4	1.2	1.8	0.5	1.5	1.1	0.4	1.5	1.8
	AP	0.2	0.7	1.9	0.3	0.9	2.0	0.4	1.1	0.9	0.3	0.7	1.1
	EOS	0.1	0.3	0.6	0.1	0.2	0.4	0.2	0.8	0.7	0.1	0.6	0.7
	EOH	0.0	0.3	0.5	0.0	0.2	0.4	0.2	0.7	0.6	0.1	0.5	0.6
	EOP	0.0	0.1	0.3	0.0	0.0	0.2	0.0	0.1	0.2	0.0	0.1	0.2
	Total cerar	2.5	6.2	12.0	3.3	7.3	13.9	4.4	11.7	10.0	3.3	9.2	13.5
Ceramide composition (%)	NDS	21.8	10.6	7.5	11.0	8.8	9.4	9.0	6.3	6.2	10.2	7.5	6.3
	NS	18.2	11.1	7.8	18.9	11.5	8.5	17.4	11.3	9.8	16.6	12.7	10.3
	NH	11.3	15.8	14.5	15.8	15.7	15.7	16.9	18.2	18.8	17.9	18.6	20.5
	NP	11.9	15.7	23.0	12.4	15.9	23.8	15.4	20.4	25.1	13.2	15.4	23.9
	ADS	2.7	1.9	1.9	3.6	2.6	2.2	2.4	1.4	1.2	2.9	1.6	1.2
	AS	12.7	8.6	5.3	13.1	9.9	6.1	9.9	6.0	4.4	11.8	7.9	4.8
	AH	7.7	14.5	12.3	10.8	16.0	12.8	11.8	13.0	10.8	13.6	15.9	13.1
	AP	9.4	11.2	16.2	10.0	12.8	14.1	9.4	9.2	8.7	9.8	8.1	8.5
	EOS	2.2	4.9	4.8	2.0	3.1	3.1	3.7	6.8	7.4	2.1	6.3	5.3
	EOH	1.4	4.7	4.4	1.5	3.3	2.8	3.7	6.3	5.7	1.8	5.0	4.8
	EOP	0.8	1.0	2.4	1.0	0.5	1.5	0.5	1.2	1.8	0.2	1.0	1.3
	non-EO	95.7	89.4	88.4	95.6	93.1	92.5	92.2	85.8	85.2	95.9	87.7	88.7
	EO	4.3	10.6	11.6	4.4	6.9	7.5	7.8	14.2	14.8	4.1	12.3	11.3
	Total	100.0	100.0	100.0	100.0	100.0	100.0	100.0	100.0	100.0	100.0	100.0	100.0
Average carbon numbers of ceramide (caluculated by mol ratio)	NDS	37.8	40.4	44.5	41.3	43.8	45.2	42.3	45.0	45.6	43.1	44.9	45.7
	NS	38.3	42.1	44.2	40.1	43.2	44.4	41.8	44.7	45.2	42.4	44.6	45.5
	NH	42.1	44.2	44.5	42.5	44.1	44.4	43.1	44.5	44.6	43.5	44.3	44.6
	NP	42.3	44.4	45.1	43.6	44.8	45.1	44.1	45.0	45.1	44.1	44.9	44.9
	ADS	39.2	40.7	41.7	40.0	41.4	42.1	41.1	41.5	41.4	41.6	41.5	41.7
	AS	37.7	39.4	41.7	39.0	40.9	41.6	39.6	40.7	41.4	40.6	41.2	42.4
	AH	42.5	42.9	43.6	42.6	43.1	43.7	42.6	43.1	43.4	43.3	43.4	43.7
	AP	40.8	43.3	43.6	42.2	43.5	43.8	43.2	43.4	43.5	43.5	43.7	43.8
	EOS	69.5	68.7	68.5	68.9	68.6	68.7	68.6	69.0	68.9	68.7	69.0	68.8
	EOH	68.8	67.6	67.7	67.7	67.5	67.5	67.4	67.9	68.0	68.0	67.6	67.8
	EOP	69.5	69.3	68.1	68.0	69.2	68.4	68.1	69.0	68.5	68.8	69.3	68.4

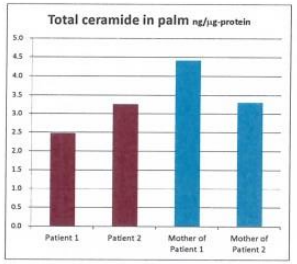

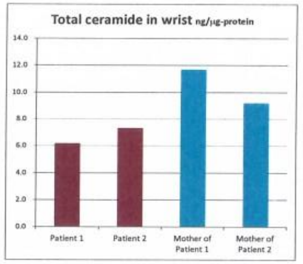

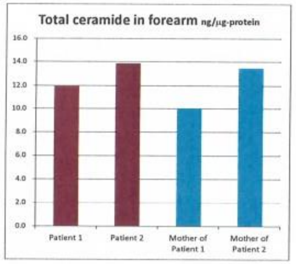

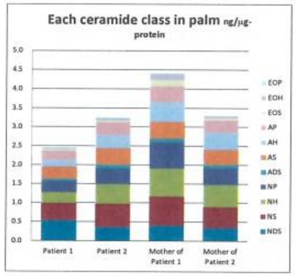

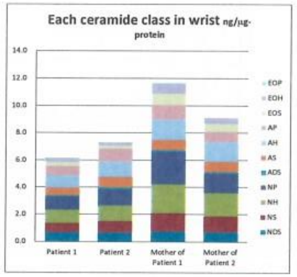

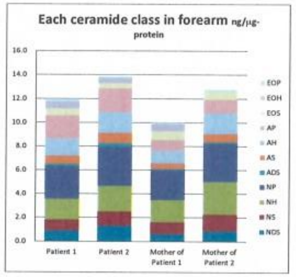

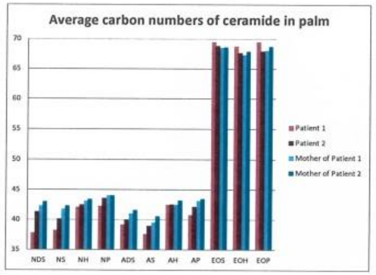

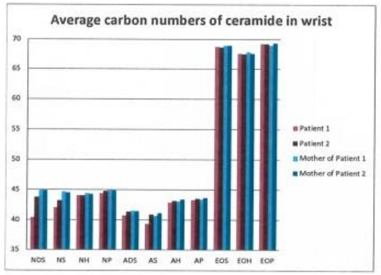

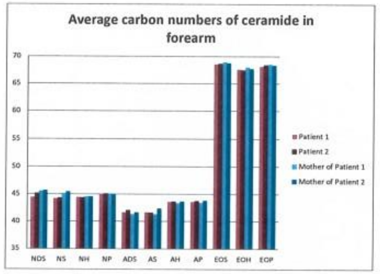

ceramide species	m/z value	retention times
NDS C32	570.5	16.5
NDS C33	584.5	17.1
NDS C34	598.5	17.7
NDS C35	612.5	18.3
NDS C36	626.5	19.1
NDS C37	640.5	19.7
NDS C38	654.5	20.5
NDS C39	668.6	21.3
NDS C40	682.6	22.0
NDS C41	696.6	22.7
NDS C42	710.6	23.4
NDS C43	724.6	24.1
NDS C44	738.6	24.9
NDS C45	752.6	25.6
NDS C46	766.7	26.3
NDS C47	780.7	26.9
NDS C48	794.7	27.6
NDS C49	808.7	28.2
NDS C50	822.7	28.8
NDS C51	836.7	29.5
NDS C52	850.7	30.0
NDS C53	864.8	30.6
NDS C54	878.8	31.5
NS C32	568.5	15.9
NS C33	582.5	16.7
NS C34	596.5	17.3
NS C35	610.5	17.9
NS C36	624.5	18.6
NS C37	638.5	19.3
NS C38	652.5	20.0
NS C39	666.6	20.7
NS C40	680.6	21.5
NS C41	694.6	22.2
NS C42	708.6	22.9
NS C43	722.6	23.6
NS C44	736.6	24.4
NS C45	750.6	25.1
NS C46	764.7	25.8
NS C47	778.7	26.4
NS C48	792.7	27.1
NS C49	806.7	27.8
NS C50	820.7	28.4
NS C51	834.7	29.0
NS C52	848.7	29.6
NS C53	862.8	30.2
NS C54	876.8	31.1
NH C32	584.5	13.6
NH C33	598.5	14.3
NH C34	612.5	14.9
NH C35	626.5	15.6
NH C36	640.5	16.5
NH C37	654.5	17.1
NH C38	668.6	17.7
NH C39	682.6	18.4
NH C40	696.6	19.1
NH C41	710.6	19.8
NH C42	724.6	20.5
NH C43	738.6	21.1
NH C44	752.6	21.9
NH C45	766.7	22.6
NH C46	780.7	23.3
NH C47	794.7	24.0
NH C48	808.7	24.7
NH C49	822.7	25.4
NH C50	836.7	26.0
NH C51	850.7	26.7
NH C52	864.8	27.7
NH C53	878.8	28.4
NH C54	892.8	29.2
NP C32	586.5	15.0
NP C33	600.5	15.6
NP C34	614.5	16.7
NP C35	628.5	17.1
NP C36	642.5	17.7
NP C37	656.5	18.4
NP C38	670.6	19.1
NP C39	684.6	19.8
NP C40	698.6	20.5
NP C41	712.6	21.2
NP C42	726.6	21.9
NP C43	740.6	22.6
NP C44	754.6	23.4
NP C45	768.7	24.1
NP C46	782.7	24.8
NP C47	796.7	25.5
NP C48	810.7	26.2
NP C49	824.7	26.9
NP C50	838.7	27.5
NP C51	852.7	28.2
NP C52	866.8	28.8
NP C53	880.8	29.4
NP C54	894.8	30.0

ceramide species	m/z value	retention times
ADS C32	586.5	15.6
ADS C33	600.5	16.6
ADS C34	614.5	17.2
ADS C35	628.5	17.8
ADS C36	642.5	18.5
ADS C37	656.5	19.2
ADS C38	670.6	19.9
ADS C39	684.6	20.6
ADS C40	698.6	21.3
ADS C41	712.6	22.0
ADS C42	726.6	22.7
ADS C43	740.6	23.5
ADS C44	754.6	24.2
ADS C45	768.7	24.9
ADS C46	782.7	25.6
ADS C47	796.7	26.2
ADS C48	810.7	26.9
ADS C49	824.7	27.6
ADS C50	838.7	28.1
ADS C51	852.7	29.0
ADS C52	866.8	29.5
ADS C53	880.8	30.1
ADS C54	894.8	30.9
AS C32	584.5	15.0
AS C33	598.5	16.1
AS C34	612.5	16.7
AS C35	626.5	17.3
AS C36	640.5	18.0
AS C37	654.5	18.6
AS C38	668.6	19.3
AS C39	682.6	20.0
AS C40	696.6	20.7
AS C41	710.6	21.4
AS C42	724.6	22.2
AS C43	738.6	22.8
AS C44	752.6	23.6
AS C45	766.7	24.3
AS C46	780.7	25.0
AS C47	794.7	25.7
AS C48	808.7	26.4
AS C49	822.7	27.0
AS C50	836.7	27.7
AS C51	850.7	28.2
AS C52	864.8	28.9
AS C53	878.8	29.9
AS C54	892.8	30.4
AH C32	600.5	13.8
AH C33	614.5	14.5
AH C34	628.5	14.9
AH C35	642.5	15.4
AH C36	656.5	15.9
AH C37	670.6	16.5
AH C38	684.6	17.0
AH C39	698.6	17.7
AH C40	712.6	18.4
AH C41	726.6	19.0
AH C42	740.6	19.7
AH C43	754.6	20.4
AH C44	768.7	21.0
AH C45	782.7	21.8
AH C46	796.7	22.5
AH C47	810.7	23.2
AH C48	824.7	23.9
AH C49	838.7	24.6
AH C50	852.7	25.3
AH C51	866.8	26.2
AH C52	880.8	27.1
AH C53	894.8	27.6
AH C54	908.8	28.7
AP C32	602.5	14.6
AP C33	616.5	15.5
AP C34	630.5	16.0
AP C35	644.5	16.6
AP C36	658.5	17.3
AP C37	672.6	17.9
AP C38	686.6	18.5
AP C39	700.6	19.2
AP C40	714.6	19.9
AP C41	728.6	20.6
AP C42	742.6	21.3
AP C43	756.6	22.1
AP C44	770.7	22.8
AP C45	784.7	23.5
AP C46	798.7	24.2
AP C47	812.7	24.9
AP C48	826.7	25.6
AP C49	840.7	26.3
AP C50	854.7	27.0
AP C51	868.8	27.7
AP C52	882.8	28.4
AP C53	896.8	28.9
AP C54	910.8	30.0

ceramide species	m/z value	retention times
EOS C66	1070.9	30.1
EOS C67	1084.9	30.5
EOS C68	1098.9	31.0
EOS C69	1112.9	31.5
EOS C70	1126.9	32.0
EOS C71	1140.9	32.4
EOS C72	1154.9	33.0
EOH C66	1086.9	28.2
EOH C67	1100.9	28.7
EOH C68	1114.9	29.2
EOH C69	1128.9	29.7
EOH C70	1142.9	30.2
EOH C71	1156.9	30.8
EOH C72	1170.9	31.4
EOP C66	1088.9	29.4
EOP C67	1102.9	30.0
EOP C68	1116.9	30.3
EOP C69	1130.9	30.8
EOP C70	1144.9	31.4
EOP C71	1158.9	31.9
EOP C72	1172.9	32.3
